# Circulating tumor DNA in patients with colorectal adenomas: assessment of detectability and genetic heterogeneity

**DOI:** 10.1038/s41419-018-0934-x

**Published:** 2018-08-30

**Authors:** Ni Ni Moe Myint, Ajay M. Verma, Daniel Fernandez-Garcia, Panchali Sarmah, Patrick S. Tarpey, Saif Sattar Al-Aqbi, Hong Cai, Ricky Trigg, Kevin West, Lynne M. Howells, Anne Thomas, Karen Brown, David S. Guttery, Baljit Singh, Howard J. Pringle, Ultan McDermott, Jacqui A. Shaw, Alessandro Rufini

**Affiliations:** 1 Leicester Cancer Research Centre, University of Leicester, Leicester, LE2 7LX UK; 2 University of Leicester, University Hospital of Leicester, Leicester, LE2 7LX UK; 3 Wellcome Sanger Institute, Hinxton, CB10 1SA UK; 4 Department of Pathology and Poultry Diseases, Faculty of Veterinary Medicine, University of Kufa, Kufa, Iraq; 5 Kettering General Hospital NHS Foundation Trust, Kettering, NN16 8UZ UK

## Abstract

Improving early detection of colorectal cancer (CRC) is a key public health priority as adenomas and stage I cancer can be treated with minimally invasive procedures. Population screening strategies based on detection of occult blood in the feces have contributed to enhance detection rates of localized disease, but new approaches based on genetic analyses able to increase specificity and sensitivity could provide additional advantages compared to current screening methodologies. Recently, circulating cell-free DNA (cfDNA) has received much attention as a cancer biomarker for its ability to monitor the progression of advanced disease, predict tumor recurrence and reflect the complex genetic heterogeneity of cancers. Here, we tested whether analysis of cfDNA is a viable tool to enhance detection of colon adenomas. To address this, we assessed a cohort of patients with adenomas and healthy controls using droplet digital PCR (ddPCR) and mutation-specific assays targeted to trunk mutations. Additionally, we performed multiregional, targeted next-generation sequencing (NGS) of adenomas and unmasked extensive heterogeneity, affecting known drivers such as *AP*C, *KRAS* and mismatch repair (MMR) genes. However, tumor-related mutations were undetectable in patients’ plasma. Finally, we employed a preclinical mouse model of *Apc*-driven intestinal adenomas and confirmed the inability to identify tumor-related alterations via cfDNA, despite the enhanced disease burden displayed by this experimental cancer model. Therefore, we conclude that benign colon lesions display extensive genetic heterogeneity, that they are not prone to release DNA into the circulation and are unlikely to be reliably detected with liquid biopsies, at least with the current technologies.

## Introduction

Colorectal cancer (CRC) is the third most common malignancy, causing over 50,000 deaths per year in the United States^1^. CRC develops from precursor adenoma lesions through two major routes. Constitutive Wnt signaling, triggered by inactivation of the tumor suppressor gene *APC*, followed by mutations in codons 12 and 13 of the *KRAS* oncogene, characterize the traditional CRC pathway originating from tubular adenomas (TAs), villous adenomas (VAs) and mixed tubulovillous adenomas (TVAs)^2^. The serrated pathway, arising from sessile serrated adenomas (SSAs), hyperplastic polyps (HPs) and traditional serrated adenomas (TSAs), is instead characterized by microsatellite instability, fostered by inactivation of the MMR, methylation of CpG islands and mutations in the *BRAF* oncogene, especially the most common glutamate to valine substitution in position 600^3^.

CRC displays remarkable therapeutic resilience, fostered by widespread genetic heterogeneity^4–6^. Cancer mutations are categorized as truncal or clonal when they originate from the initial tumor-founder clone and are ubiquitous throughout the neoplasia, whereas mutations present only in a subset of tumor regions are known as subclonal or private^7^. The heterogeneity of CRC is shaped by neutral evolution, with mutations spreading in the absence of Darwinian selection and clonal sweeps^4,5^, but its origin remains controversial. The “Big Bang” model suggests that the majority of somatic alterations arise during the initial stages of tumorigenesis, ahead of the emergence of detectable adenomas^4^. Accordingly, most private mutations are pervasive throughout the neoplasia because their accumulation occurs swiftly after the establishment of an initial founder clone that harbors all truncal mutations. However, the monoclonal origin of CRC has been brought into question by genetic analysis of individual colonic crypts of benign adenomas, in which polyclonality of driver events, including distinct mutations in *APC* and *KRAS*, has been identified^8,9^.

Survival of patients with CRC depends on tumor stage at diagnosis. Removal of adenomas and stage I cancers through polypectomy at colonoscopy and/or surgery is largely curative^10^. In order to improve diagnosis of early cancers, in England, the Bowel Cancer Screening Programme (BCSP) offers guaiac Fecal Occult Blood test (gFOBt) biennially to individuals aged 60 to 74 years. The BCSP has improved the detection rate of early-stage cancers and has reduced mortality^11–13^. However, the test has limited sensitivity for right-sided tumors^12,13^ and for benign adenomas^14–16^, and the rate of false-positive and negative results is noteworthy^13^. The test also suffers modest uptake of around 50%^13,17^. In an attempt to improve detection rate and compliance, the BCSP will replace gFOBt with the Fecal Immunochemical Test, which has improved sensitivity and diagnostic accuracy, all from a single stool sample, as opposed to the multiple sampling required with gFOBt^15,18,19^.

One attractive possibility to improve detection of adenomas and compliance is the implementation of diagnostic tests that exploit analysis of cell-free DNA (cfDNA). Dying cells release fragmented DNA into the circulation, and in cancer patients the fraction of cfDNA that originates from tumor cells (circulating tumor DNA (ctDNA)) carries tumor-related alterations, which can be detected using next-generation sequencing (NGS) and PCR-based methodologies^20^. Analysis of cfDNA, known as liquid biopsy, is cost effective, minimally invasive, and its specificity can be enhanced by tailoring the analysis towards the detection of tumor-specific mutations^21^. Recently, liquid biopsies have been used to detect minimal residual disease and track recurrence after surgical resection of localized disease. Patient-specific PCR assays successfully amplified somatic aberrations in serial plasma samples of post-operative CRC patients (stages I–IV), enabling identification of relapses with an average lead time of 10 months before clinical manifestation of the disease^22^. Consistently, postsurgical detection of ctDNA successfully predicted recurrence after surgical removal of stage II CRC^23^. In contrast, the application of liquid biopsies for detection of benign tumors has proved challenging^24,25^. The probability of detecting ctDNA is low in early-stage CRC^25^ and different groups have reported dissimilar results with regard to the diagnostic value of total cfDNA levels or analysis of KRAS mutations in plasma of patients with adenomas^26–29^. Encouraging studies reported an increase in total cfDNA or even detected tumor-related mutations in patients with benign adenomas^26,28^. Notwithstanding their validity, these studies employed clinically impractical technologies, such as radioactive-based dot-blot hybriditation^28^, or analyzed patient cohorts comprising large adenomas^26^. Therefore, the wider applicability of ctDNA analysis for detection of low-grade, benign lesions is yet to be ascertained.

Several parameters might influence the detection rate of benign lesions. Adenomas are typically small and do not manifest the sustained apoptosis or necrosis observed in advanced cancers. Moreover, tumor heterogeneity can affect cfDNA analysis, as plasma representation of trunk alterations is higher compared to subclonal mutations^30–32^. Intriguingly, heterogeneity has been described in adenomas, which also affects *KRAS*^8,9^, suggesting that this oncogene might be subclonal and therefore inadequate for targeted cfDNA testing. Notwithstanding, the advent of highly sensitive technologies, such as droplet digital PCR (ddPCR), provides a cost-effective and efficient way to identify low-frequency DNA mutations in plasma, enabling more effective testing of the diagnostic usefulness of plasma DNA analysis for early disease detection.

**Fig. 1:**
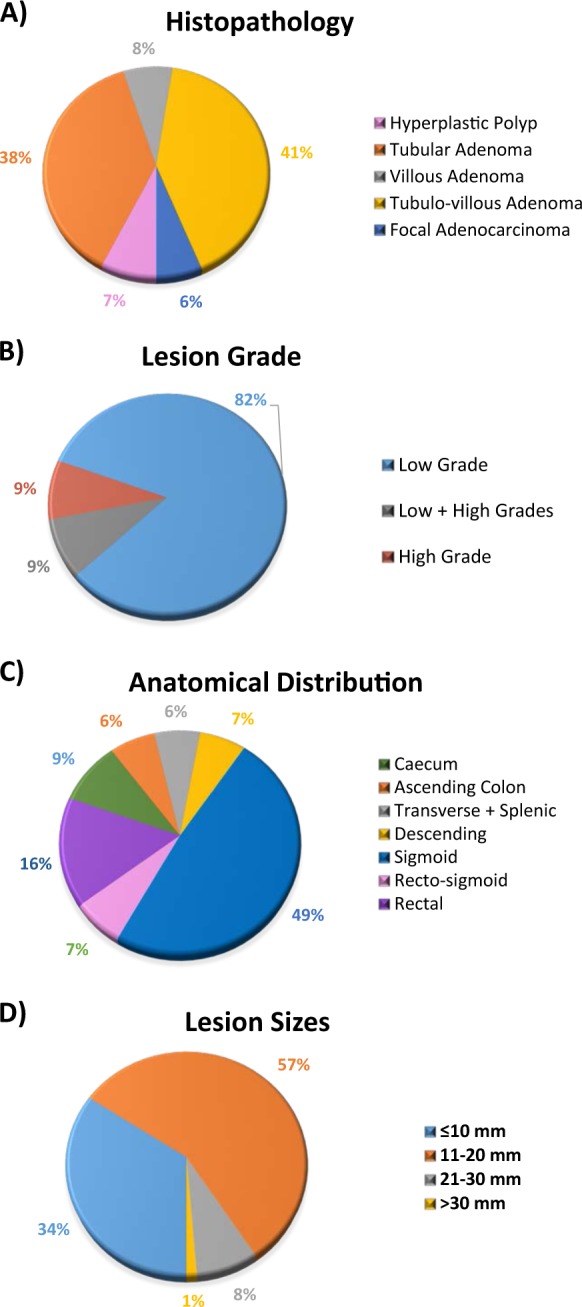
Clinicopathological information of patient samples. All data were obtained from histology reports for the 76 patient cases. **a** Distribution of different histological subtypes of adenomas. A small portion of lesions (6%) showed focal adenocarcinoma (AC) within adenomatous regions. **b** Pathology assessment showed that most were low-grade lesions (82%). **c** Half of the lesions were found in the sigmoid colon (49%), with a significant portion located in the rectum (16%). The rest were distributed throughout the colon. **d** Longest measurements of diameter listed on histology report were used; measurements of FFPE sections were used as proxy when a report did not state any measurements

Here, we assessed a cohort of individuals recruited through the BCSP (Fig. 1) to test the feasibility of cfDNA analysis for the detection of benign colorectal adenomas. To that end, we searched for significant changes in total cfDNA and for tumor-related mutations in the plasma of patients with adenomas, using ddPCR and mutation-specific assays tailored to trunk mutations identified by multiregional targeted NGS of adenoma tissues. While changes in cfDNA or mutations were not detectable, NGS analysis unmasked extensive genetic heterogeneity of colonic adenomas, affecting known drivers such as *AP*C, *KRAS* and MMR genes. Finally, using a mouse model of *Apc*-driven intestinal adenomas, we confirmed that, despite enhanced tumor burden, no alterations could be identified in the cfDNA.

## Results

### Assessment of *BRAF* and *KRAS* mutations in adenomas

DNA from 76 formalin-fixed, paraffin-embedded (FFPE) adenoma specimens, mostly low-grade and small lesions (Fig. 1), was tested to identify mutations in *KRAS* and *BRAF* oncogenes. BRAF has been reported to be mutated in 10% of CRC and to be the main driver of the serrated pathway, whereas KRAS mutations are mutually exclusive to BRAF and affect 40% of CRC^33,34^. With the exception of the commercially available *KRAS-c*.*38* mutation assay for ddPCR, *BRAF* and *KRAS* mutations were detected employing degenerate assays developed in-house (see Materials and methods). Hence, for each sample, four assays were run to check for *BRAF-c*.*1799*, *KRAS-c.34* and *KRAS-c.35* mutations by quantitative PCR (qPCR), and the *KRAS-c.38G*>*A* substitution by ddPCR. Wild-type (WT) allele PCR was run in parallel and samples were considered positive when mutations were detected at a fractional abundance above 1%.

We identified 39 cases (51%) bearing mutations in either *KRAS* or *BRAF*. The *BRAF-c.1799* hotspot mutations were identified in 4 out of 76 cases (5%), a frequency lower than what has been reported^33,34^ (Supplemental Fig. 1), but in agreement with other published data^35^. All four *BRAF*-positive cases were from the recto-sigmoid region, with 3 classified as HPs and 1 as a low-grade VA. *KRAS* mutations were identified in 35 out of 76 cases (46%) (Table 1). Among these *KRAS*-positive cases, mutations at c.35G were the most common with 27 cases (42%), including 11 TVA cases, 8 TAs, 4 VAs and 1 benign HP. Mutations at codon 34 were detected in 8 cases (10.5%), including 4 TVA cases, 2 TA adenomas and 1 VA lesion (Table 2). Finally, mutations at the base c.38G were found in 9 cases (22.4%): 7 TVAs cases and 2 TAs (Table 3). Interestingly, most patients showed varying degrees of fractional abundance of the mutated alleles. This variability could depend on contamination from the stromal tissue, since tumor enrichment by microdissection was not performed in this experiment, but it could also signify heterogeneity of the mutant oncogenes. The latter possibility was confirmed by the identification of double *KRAS* mutations in the same adenomas in over 10% of patients (9/76). Indeed, 5 adenomas showed simultaneous mutations in codons 35 and 38, in 3 specimens both the 34 and 35 hotspots were mutated, and in 1 patient specimen codons 34 and 38 were both affected. Double mutations affecting *KRAS* codons 12 and 13 were already observed in colon neoplasia^8,36^. The simultaneous presence of multiple mutations in *KRAS* suggests tumor heterogeneity in early adenomas.

**Table 1:** Histology details of *KRAS*-*c.35G* mutant adenomas

Sample ID	No. of FFPE blocks	% Fractional abundance in FFPE samples	Histology	Polyp dimensions (mm)
H146	1	9.4001	Transverse (benign HP)	8 × 5 × 4
H151	1	3.1402	Splenic flexure (LG TA)	5 × 4
H154	4	0.0000–10.8343	Descending-sigmoid (LG TA/TVA)	17 × 15 × 14
H185	1	1.2587	Sigmoid (LG TVA)	10 × 8 × 17
H189	1	18.612	Rectal (LG TA)	12
H263	11	0.0000–6.2256	Rectal (LG+HG TVA, focal AC)	17 × 30 × 10−30 × 25 × 22
H265	13	19.5678–37.4255	Distal sigmoid (LG+HG VA)	55 × 35 × 20
H266	9	12.9668–26.7434	Sigmoid (LG VA)	N/A
H321	1	0.9613	Colonic (LG+HG TVA)	15 × 7
H325	1	1.0923	Colonic (LG TVA)	20 × 14 × 8
H329	2	4.5123–9.0246	Rectal (LG VA)	11 × 9 × 9−14 × 9 × 7
H333	2	6.2192–8.3315	Recto-sigmoid (LG TA)	19 × 16 × 10
H334	2	0.0000–1.5870	Recto-sigmoid (LG TA+TVA)	7 × 9 × 4–12 × 10 × 4
H335	2	2.8364–8.9973	Sigmoid-cecal (LG TVA)	10–12
H336	2	5.3522–11.8328	Sigmoid (LG TVA)	14
H339	2	0.0005–1.2086	Recto-sigmoid (LG TA)	6 × 5 × 5−12 × 8 × 8
H343	2	0.7317–1.3000	Sigmoid (LG TVA)	15 × 10 × 15
H344	2	2.1852–2.8104	Colonic (LG TVA)	20 × 20 × 10
H345	2	0.0016–1.5049	Descending-sigmoid (LG TA)	7 × 5 × 4–11 × 9 × 7
H346	2	0.0003–2.5250	Ascending-rectal (LG TA)	12−15
H347	2	0.7243–1.8001	Recto-sigmoid (LG TVA)	21 × 15 × 10
H693	2	0.3259–1.6247	Cecal (LG TA)	N/A
H696	2	7.0348–7.4390	Ascending (LG TVA)	N/A
H697	1	5.4522	Cecal (LG TA)	10 × 4 × 2
H774	2	9.3524–18.4350	Cecal (AC Duke’s B)	N/A
H783	2	13.9502–14.6877	Distal descending (HG TVA with focal AC)	N/A
H784	2	18.9137–23.7891	Colonic (polyp–HG VA, AC–invasive, pT1)	N/A

For cases where more than one FFPE blocks were tested, lowest and highest mutation frequencies are reported

*HP* hyperplastic polyp, *LG* low-grade, *HG* high-grade, *TA* tubular adenoma, *VA* villous adenoma, *TVA* tubulovillous adenoma, *MD* mild

**Table 2:** Histology details of *KRAS-c.34G* mutant adenomas

Sample ID	No. of FFPE blocks	% Fractional abundance in FFPE samples	Histology	Polyp dimensions (mm)
H183	1	0.9667	Mid transverse (LG TA)	10 × 6
H314	1	2.7618	Distal sigmoid (LG TA)	16 × 7 × 5
H325	1	1.0923	Colonic (LG TVA)	20 × 14 × 8
H329	2	0.0006–5.3010	Rectal (LG VA)	11 × 9 × 9 14 × 9 × 7
H338	2	0.7336–3.5509	Sigmoid (LG TVA)	16 × 15 × 10
H344	2	0.0000–1.9799	Colonic (LG TVA)	20 × 20 × 10
H775	1	11.093	Colonic (MD AC, Duke’s C1)	N/A
H785	1	9.0330	Recto-sigmoid (sessile HG TVA, focal pT1 AC)	16 × 11 × 9

Histology and polyp dimensions were obtained from the histology reports. For cases where more than one FFPE blocks were tested, lowest and highest mutation frequencies are reported

*LG* low-grade, *HG* high-grade, *TA* tubular adenoma, *VA* villous adenoma, *TVA* tubulovillous adenoma, *MD* mild differentiated, *AC* adenocarcinoma

**Table 3:** Histology details of *KRAS*-*c.38G* mutant adenomas

Sample ID	No. of FFPE blocks	% Fractional abundance in FFPE samples	Histology	Polyp dimensions (mm)
H142	1	16.3347	Sigmoid (LG TVA)	17 × 10 × 15
H149	8	0.0000–3.8205	Sigmoid (LG TVA)	15 × 15 × 15–25 × 20 × 25
H151	1	5.0351	Splenic flexure (LG TA)	5 × 4
H183	1	6.2698	Mid transverse(LG TA)	10 × 6
H190	1	30.4348	Recto-sigmoid (LG TVA)	14
H334	2	0.0000–4.6363	Recto-sigmoid (LG TA+TVA)	7 × 9 × 4–12 × 10 × 4
H335	2	0.0565–1.4967	Sigmoid-cecal (LG TVA)	10–12
H336	2	2.3732–7.1057	Sigmoid (LG TVA)	14
H347	2	0.0500–2.1894	Recto-sigmoid (LG TVA)	21 × 15 × 10

For cases where more than one FFPE blocks were tested, lowest and highest mutation frequencies  are reported

*LG* low-grade, *HG* high-grade, *TA* tubular adenoma, *VA* villous adenoma, *TVA* tubulovillous adenoma, *MD* mild differentiated, *AC* adenocarcinoma

### Quantification of total cfDNA levels

**Fig. 2:**
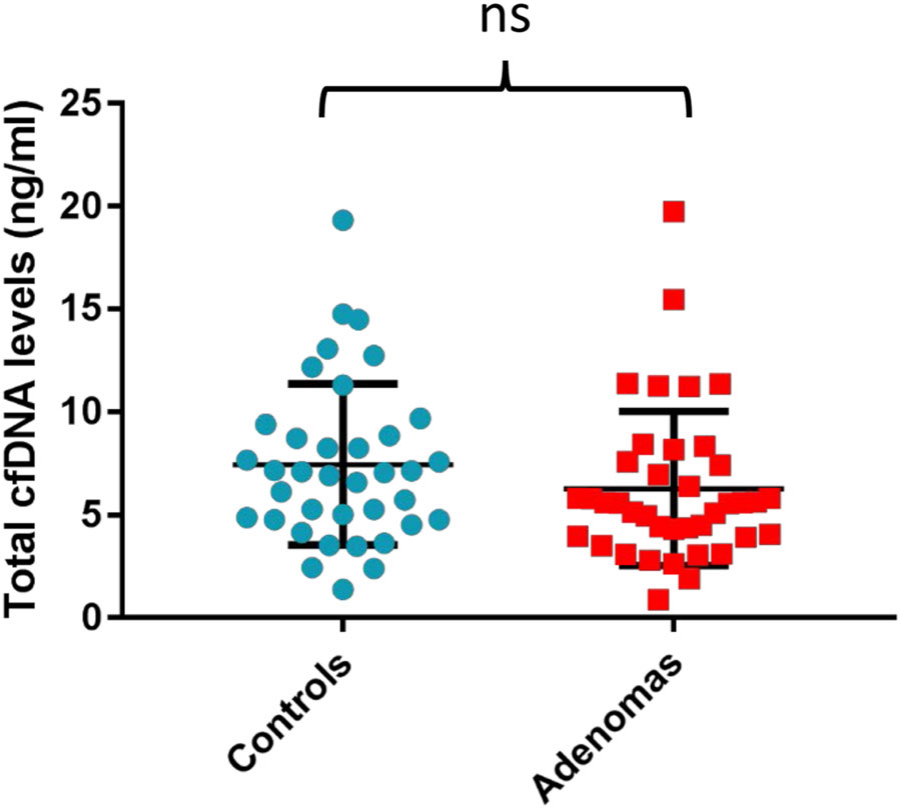
Comparison of total plasma cfDNA levels in the patient and control groups. Comparing total plasma cfDNA levels in the patient (*n* = 39) and the control (*n* = 37) groups. Each dot represents one sample. Horizontal bars represent mean ± SD. The mean total cfDNA levels were 7.456 ± 0.642 ng/mL for the control group and 6.285 ± 0.599 ng/mL for the patient group (*p* = 0.186, two-tailed *t*-test)

The cases that were positive for at least one *BRAF* or *KRAS* mutation were selected for quantitative analysis of the total amount of cfDNA to test the hypothesis that elevated cfDNA levels are associated with the presence of early neoplastic lesions^26^. The control group consisted of 37 individuals who had a negative colonoscopy outcome following referral for a false-positive gFOBt result. The mean total cfDNA concentration was slightly higher in the control group (7.5 ± 0.64 ng/mL) compared to the patient group (6.3 ± 0.6 ng/mL), but this difference was not statistically significant (*p* = 0.19; Fig. 2). The cfDNA yield in the control group did not show any overt deviation from control cohorts described in other studies^26,37^. On the other hand, the cfDNA levels in the adenoma group were lower than previously reported^26^. The inclusion of patients with early, small-sized adenomas in our analysis might explain, at least partially, the inability to identify any increase in cfDNA. Notwithstanding, our data indicate that no discernible alteration in total plasma cfDNA concentrations exists in patients with precancerous lesions compared to healthy subjects. A lack of significant increase in total plasma cfDNA levels in individuals with premalignant colon lesions has also been reported by others^29^.

### Analysis of *KRAS* and *BRAF* mutations in plasma

**Fig. 3:**
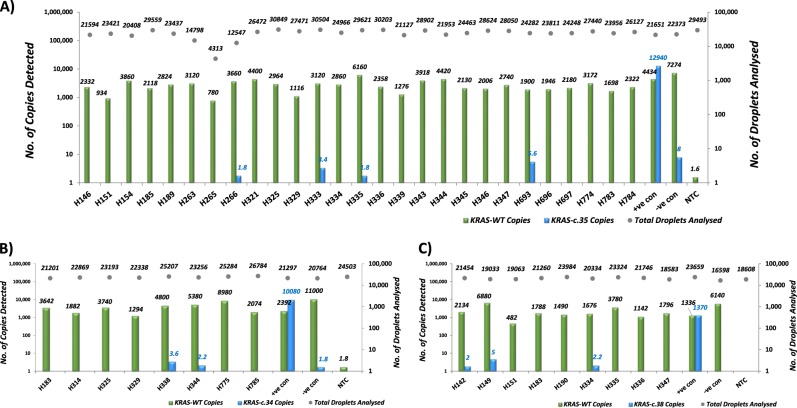
Assessment of *KRAS* mutations in plasma samples. Bar charts show merged data from two runs for each patient sample. The number of WT (green bars) and mutant (blue bars) copies detected are shown on left *y*-axis. The total number of droplets analyzed (gray dots) are indicated on right *y*-axis. **a** *KRAS-c.35*; **b** *KRAS-c.34*; **c** *KRAS-c.38*. The “+ve con” indicates positive control from 10 ng of genomic DNA isolated from SW480 (c.35G>T/p.G12V) CRC cell line, A549 (c.34G>A/p.G12S) lung cancer cell line, and HCT116 (c.38G>A/p.G13D) CRC cell line. The “–ve con” indicates negative control from 10 ng of hgDNA. NTC no template control

The lack of an increase in plasma cfDNA levels does not necessarily exclude the possibility that tumor-specific mutations might be detected in the cfDNA. Therefore, we sought to investigate whether *BRAF* and *KRAS* mutations identified in the FFPE specimens were represented in the matched plasma samples. First, we assessed the sensitivity of mutation assays using ddPCR and found that mutant DNA was detectable below a 0.5% allele frequency (Supplemental Fig. 2). Then, using ddPCR, we tested matched plasma samples from 4 *BRAF* mutant cases, 8 *KRAS-c.34G-*positive cases, 27 *KRAS-c.35G*-positive cases and 9 patient cases bearing the *KRAS-c.38* (Fig. 3). Variable copy numbers of WT *KRAS* and *BRAF* genes were detected and numerous patients had robust representation of the WT DNA. However, we were unable to identify with confidence mutant DNA copies in the circulating DNA in all the cases analyzed. Indeed, the sporadic positive samples showed a limited number of droplets and fractional mutations below the minimum value predicted by the Poisson distribution analysis (not shown). For example, with regard to *KRAS-c.35*, 4 samples showed marginal positivity for the mutant alleles (Fig. 3a), over a range of fractional abundances (from 0.029% in sample H335 to 0.294% in H693). Poisson analysis predicted detection of 0.0–0.6 mutant copies at the minimum threshold (not shown), pointing towards random positivity. Indeed, when positive samples were retested, all but one failed to confirm the initially identified mutations. Overall, these results indicate that ctDNA in adenoma patients is not reliably detectable by ddPCR.

### Multi-regional targeted sequencing of adenomas

Data from FFPE tissue suggested that the *BRAF* and *KRAS* mutations were present at low mutational fraction in most cases. We cannot formally rule out that such poor representation of mutant DNA depends on stromal dilution of the tumor cells. However, it is likewise reasonable that genetic heterogeneity defines colon adenomas and that *KRAS* and *BRAF* mutations are subclonal. Moreover, the private nature of these oncogenic alterations might have contributed to their absence from the patients’ plasma. To assess the degree of heterogeneity in early colon lesions, six adenoma specimens (H149, H154, H263, H264, H265 and H266) (Supplemental Table 1) were selected for multiregional targeted NGS. Sequencing was carried out using an in-house custom targeted panel, which includes the most common alterations in CRC, including hotspot mutations in 116 genes, 22 genes recurrently amplified/deleted, 51 copy number regions, 121 microsatellite regions and 2 *RSpondin* gene fusions. Prior to sequencing, the adenomas were analyzed by a pathologist to identify the tumor area for macroscopic enrichment (Supplemental Table 1). DNA aliquots were quantified using the described *ALU* real-time PCR assay and approximately 200 ng of purified DNA per sample was sequenced on the Illumina NGS platform. The average sequencing depth obtained was 696× (minimum 461×, maximum 1059×).

**Fig. 4:**
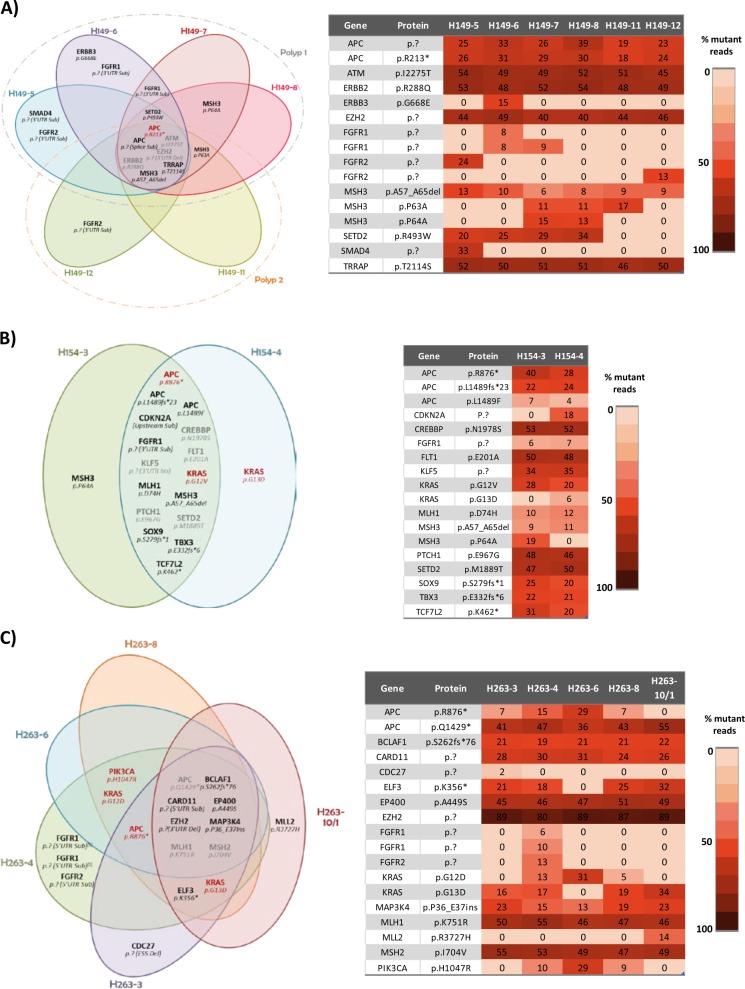

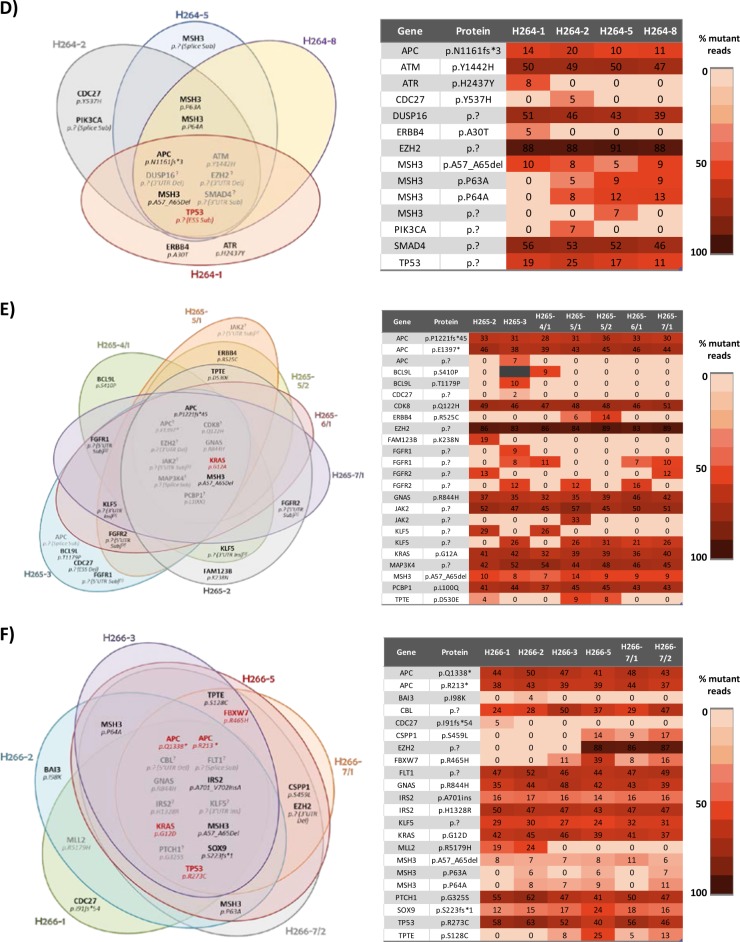
Summary of multiregional sequencing data. Venn diagrams illustrate the spatial distributions of mutations in the different sequenced regions of the indicated patients. Each colored oval represents one sequenced region. Mutations in red are driver mutations as determined by the in-house calling algorithm. The heat maps show the percentage of mutant reads for each mutation. **a** Data for case H149, **b** data for case H154, **c** data for case H263, **d** data for case H264, **e** data for case H265 and **f** data for case H266

As shown in Fig. 4, all the sequenced samples revealed widespread tumor heterogeneity, affecting various driver genes among which, remarkably, were alterations in MMR genes. Often non-ubiquitous mutations were identified in several spatially distinct tumor regions, a behavior compatible with the pervasive nature of private mutations described in the “Big Bang” model of tumorigenesis^4^.

With regard to *KRAS*, in some cases oncogenic mutations were ubiquitous (see for example c.35G>C/p.G12A for patient H265, or the c.35G>A/p.G12D mutation in case H266; Fig. 4e, f), suggesting that these might be truncal variants. However, in other adenomas *KRAS* substitutions displayed remarkable heterogeneity. In case H263, the neoplasia harbored two *KRAS* mutations: 3 out of 5 sequenced regions were positive for the c.35G>A/G12D mutation at 5–31% frequencies, and 4 out of 5 regions were positive for the c.38G>A/p.G13D mutation at 16–34% frequencies (Fig. 4c). Similarly, heterogeneity was identified in case H154 (Fig. 4b), where two spatially separated adenoma regions were sequenced and again two distinct *KRAS* mutations were detected (c.34G>T/p.G12V and c.38G>A/p.G13D). The c.38G>T substitution was detected only in one tumor region and at a very low frequency (6%), indicating that the mutation is present in a relatively small clone. We were able to confirm the *KRAS* mutational status using PCR analysis of the different regions selected for NGS (Supplemental Table 2-4).

**Fig. 5:**
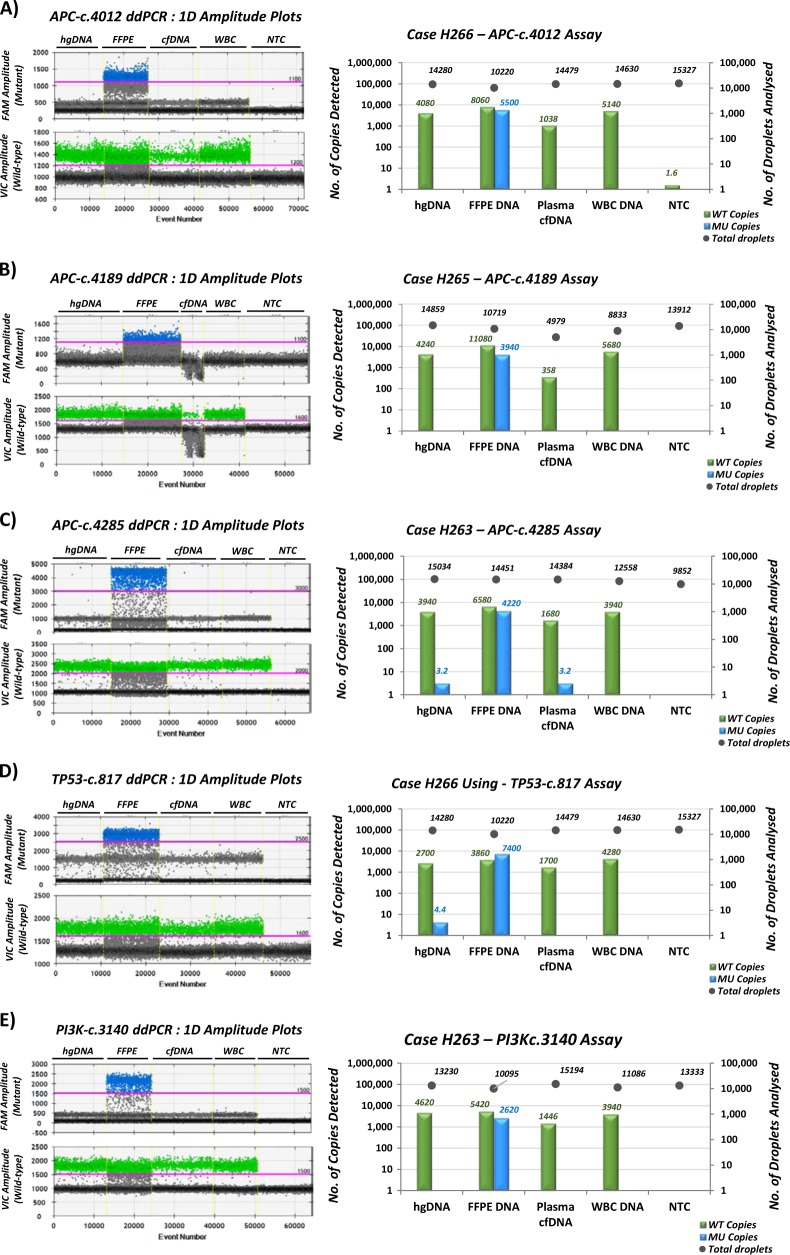
Analysis of patient-specific mutations using ddPCR. For each assay, one-dimensional (1D) amplitude plots are shown on the left with the associated manual threshold gating (pink line) for VIC (WT, green) and FAM (mutant, blue) amplitudes. On the right, the bar graphs plot the number of WT copies (green bars) and mutant copies (blue bars) detected on the left *y*-axis. Number of total droplets analyzed (gray dots) are shown on the right *y*-axis. **a** Case H266 *APC-c.4012C>T* (p.Q1338*) mutation; **b** Case H265, *APC-c.4189G>T* (p.E1397*) mutation; **c** Case H263, *APC-c.4285C>T* (p.Q1429*) mutation; **d** Case H266, *TP53-c.817C>T* (p.R273C) mutation; **e** Case H263, *PI3K-c.3140A*>*G* (p.H1047R) mutation. For each sample, 10 ng hgDNA was used as a negative control, 10 ng of matched FFPE tissue DNA was used as a positive control and 10 ng of matched white blood cell (WBC) DNA was included as a control for germline polymorphic variants. NTC no template control

Next, we used ddPCR to confirm 5 additional patient-specific mutations in FFPE samples. We also assessed DNA isolated from matched white blood cells, to exclude the possibility of germline variants, and we analyzed plasma cfDNA to test whether ddPCR assays might detect early adenomas. We selected the *APC* c.4285C>T (p.Q1429*) mutation and the *PIK3CA* c.3140A>G (p.H1047R) mutation in patient H263. The *APC* mutation at codon 1429, a non-sense substitution, resulting in a STOP codon, was identified at high frequencies in all five sequenced samples, suggesting that this was a truncal variant. We selected another *APC* mutation, c.4189G>T (p.E1397*) from case H265, which also appeared truncal. The remaining two targets were chosen from case H266: *APC (*c.4012C>T/p.Q1338*) and *TP53* (c.817C>T/p.R273C). Both targets were ubiquitous and displayed high mutational frequencies (41–50% for APC-Q1338* and 40–63% for *TP53-R273C*). ddPCR probe-based assays were designed and validated for each mutation (Supplemental Figs. 3-7), and then tested to confirm the NGS data in FFPE-derived DNA and, potentially, in plasma cfDNA. All the five mutations were confirmed to be tumor-specific alterations and were not detected in white blood cells. However, despite selecting for ubiquitous alterations, we were unable to detect any mutation in the plasma-derived DNA (Fig. 5).

### cfDNA dynamics in a mouse model of colon adenomas

**Fig. 6:**
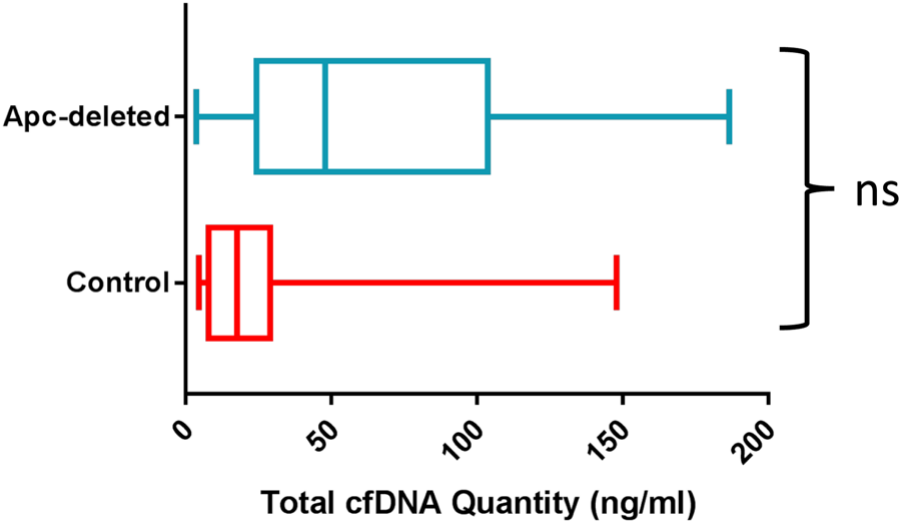
Total cfDNA quantities in the plasma of control and *Apc*-deleted mice. The plasma cfDNA load was quantified using in-house-developed mouse *Gapdh* assay. Each sample was run in duplicates. The box and whisker plots compare total cfDNA loads between two study groups. *P* = 0.092, Mann–Whitney test (*n* = 8 control and *n* = 7 *Apc*-deleted mice)

Our inability to detect cfDNA alterations from benign adenomas might reflect modest cfDNA release and/or insufficient tumor burden. To investigate whether enhanced tumor burden could enable detection of cfDNA, we employed the *Lgr5-EGFP-IRES-Cre*^*ERT2+/0*^*;Apc*^*fl/fl*^ (*Lgr5Cre*^*ER*^*-Apc*^*fl/fl*^) mouse model of colon tumorigenesis^38^. In these animals, tamoxifen-inducible *Cre*^*ER*^ recombinase enables the deletion of the *loxP*-floxed exon 14 of *Apc* (*Apc*^*fl/fl*^) in the Lgr5-positive crypt intestinal stem cells, triggering activation of the Wnt pathway and development of multiple adenomas^38–40^. A total of 17 mice (10 females, 7 males) aged 9–12 months were tested. The *Apc*-deleted group consisted of 8 tamoxifen-injected *Lgr5Cre*^*ER*^*-Apc*^*fl/fl*^ mice. The control group consisted of 6 tamoxifen-injected *Apc*^*fl/fl*^ mice (with no expression of the Cre recombinase) and 3 vehicle-injected *Lgr5Cre*^*ER*^*-Apc*^*fl/fl*^ animals. As expected, *Apc*-deleted mice developed multiple neoplastic lesions (between 4 to 36 macroadenomas, Supplemental Figure 8). To analyze the levels of total cfDNA in these animals, we used an in-house-developed assay targeting the mouse *Gadph* pseudogenes^41,42^. Plasma samples from the *Apc*-deleted trended towards increased levels of total cfDNA (66.74 ng/mL), compared to the controls (31.22 ng/mL), but without reaching statistical significance (Fig. 6). To determine whether tumor-specific alterations could be detected in the plasma of the *Apc*-deleted mice, we designed and validated ddPCR assays for identification of the recombined and non-recombined *Apc*^*fl/fl*^ alleles (Supplemental Figure 9 and Supplemental Methods). The assays were specific for the different alleles (Supplemental Figure 10A) and enabled detection of the recombined *Apc* gene in the intestinal tissue of tamoxifen-injected *Lgr5Cre*^*ER*^*-Apc*^*fl/fl*^ mice (Supplemental Figure 10B). However, we were unable to detect recombined *Apc* in the matched cfDNA (Supplemental Figure 10C). To further validate our *Apc* allele assays, in a second experiment we collected fecal material on a weekly basis, before plasma collection at endpoint. Our ddPCR assays succeeded in detecting increased fractional abundance of recombined *Apc* allele in the stool DNA (Supplemental Figure 10D), but not in plasma cfDNA (not shown).

Overall, these data indicate that, despite enhanced tumor burden, ctDNA analysis is unable to identify benign colon lesions.

## Discussion

The implementation of liquid biopsies for detection of early cancer remains challenging^21^. Many studies have provided evidence of cfDNA utility in detecting localized cancers or identifying minimal residual disease and predicting risk of recurrence in postsurgical CRC patients with stage I–IV disease^22,23,43^. However, efforts to detect benign lesions using liquid biopsies have yielded contrasting results^26,29^. Here, we have used orthologous approaches to investigate the usefulness of cfDNA testing in patients with benign polyps recruited to the BCSP. We initially measured the levels of total cfDNA, under the auspices that higher levels of cfDNA could discriminate adenoma patients from healthy controls. However, we did not detect significant differences between the two groups. These data contrast with reports by others which suggest that changes in cfDNA reflect the presence of adenomas^26^. The reasons behind this discrepancy are unclear; variations in sample storage, processing and methodologies might be responsible for the different results. We also employed a ddPCR platform to seek *KRAS* and *BRAF* mutations in the plasma of patients bearing mutated adenomas. Again, despite the high sensitivity of our ddPCR assay (<1%), we were unable to confidently identify circulating mutant DNA in our patient cohort. However, we cannot exclude that more elaborate technologies, such as BEAMing (beads, emulsion, amplification, and magnetics), CAPP-Seq (cancer personalized profiling by deep sequencing) or CUT-PCR (CRISPR-mediated, Ultrasensitive detection of Target DNA-PCR)^44–48^, could be successful with such challenging plasma samples.

Notably, when assessing *KRAS* mutational status in tumors, we frequently detected low mutational fractions and observed the coexistence of multiple *KRAS* mutations in the same patient sample. Multiregional sequencing confirmed widespread genetic heterogeneity in six adenoma samples, which affected known driver genes and confirmed the heterogeneous nature of *KRAS* mutations. This finding is of relevance, considering that similar results have been reported in the analysis of single crypts isolated from benign human adenomas, where multiple *KRAS* mutations have been detected, leading to the hypothesis of a polyclonal origin of CRC^8^. However, genetic analysis of established CRC indicates that *KRAS* mutations are truncal in nature and putatively acquired within an initial founder clone^4,5^. This apparent conundrum awaits explanation. Perhaps sampling of carcinomas has so far been too limited to identify heterogeneous *KRAS* mutations in relatively small clones, or, alternatively, during the progression from adenomas to carcinoma a clonal sweeping event might establish a founder *KRAS* mutant clone. Of note, under therapeutic selective pressure, the emergence of distinct *KRAS* mutations has been reported in the plasma of CRC patients with ensuing resistance to anti-EGFR therapy^49^. Regardless, the widespread heterogeneity of colorectal lesions is a serious deterrent to any therapeutic approach and emphasizes the necessity of developing effective strategies for the early detection and primary prevention of intestinal cancers^50,51^.

We also exploited NGS data to design patient-specific assays targeting trunk mutations for analysis of cfDNA, to leverage on experimental evidence showing higher abundance of trunk mutations in plasma^30–32^. However, we were unable to validate any mutations in the plasma-derived DNA. This consistent inability to identify tumor-related changes in cfDNA exposes the reluctance of benign lesions to shed DNA into the circulation. Of note, in postsurgical patients, plasma tumor mutations can be detected and monitored in the absence of clinical manifestations of the disease^22,23,31,52–55^, suggesting that advanced malignancies shed DNA even in the presence of low tumor burden. To better assess the relationship between benign tumors and cfDNA, we employed the *Lgr5Cre*^*ER*^*-Apc*^*fl/fl*^ mouse model of intestinal neoplasia, which develops multiple adenomas following constitutive activation of the Wnt pathway in the intestinal stem cells^38^, enabling us to evaluate the impact of increased tumor burden on the dynamics of cfDNA. Similarly, we failed to observe significant changes in total cfDNA in mouse plasma, confirming our results in human samples. Moreover, we designed ddPCR assays to detect the specific recombined *Apc* locus produced through Cre-mediated recombination, to serve as a surrogate biomarker of cancer-related mutations. With this approach we demonstrated that recombined DNA can be detected in DNA isolated from intestinal tissue and stool, but again our efforts to detect mutant DNA copies in plasma proved unsuccessful.

In summary, we report widespread genetic heterogeneity within colon adenomas and show that this heterogeneity affects genes and pathways functionally associated with the development of CRC, such as *APC*, *KRAS* and the MMR. Moreover, after thorough testing of human and mouse plasma samples, we argue that benign lesions do not release significant quantities of DNA in the circulation and are therefore unlikely to be amenable to diagnosis with liquid biopsies, at least using current technologies.

## Materials and methods

### Patients

Colonoscopy, surgical resection and blood sample collection were carried out at the Glenfield Hospital, Leicester, UK, as part of the national BCSP. All patients consented for their biological samples to be used in the study “Biomarkers for Bowel Disease (2011)” (UHL study 11005; NREC 10/H0408/116). The study received ethical approval from the Bowel Cancer Screening Programme Research Committee and Ethics Approval UHL 11005. Venous blood samples (15–20 mL) were collected 1–4 h prior to colonoscopy or surgical resection, processed within 2 h of collection and stored at −80 °C. Out of 131 patient cases, 76 patient cases (58 males, 18 females; age 57–87 years, average 67.5 years) presented with precancerous lesions (Fig. 1). For cases with multiple FFPE blocks, all available samples were included for analysis. The control group consisted of 37 individuals (22 males, 15 females; age 60–74 years, average 64.8 years) who participated in the program but showed no clinically observable polyps following false-positive gFOBt results. Additional diagnosis was noted for 8 individuals in the control group: 4 had diverticulosis, 3 had ulcerative colitis and 1 had angioectasia.

### DNA extraction from FFPE tissue and human plasma

For the FFPE samples, DNA was extracted from a minimum of three 4 μm FFPE sections per sample using GeneRead^TM^ DNA FFPE tissue kit (Qiagen®). Sections were de-waxed in xylene and re-hydrated by serial immersions in Industrial Methylated Spirit (IMS) before DNA extraction was carried out as per the manufacturer’s instructions. For the plasma samples, DNA was extracted from 2.5–3 mL plasma per patient using QIAamp® Circulating Nucleic Acid kit (Qiagen®). Samples were stored at −80 °C and thawed to room temperature prior to the extraction. The manufacturer’s protocol for the extraction using a QIAvac 24 Plus vacuum manifold (Qiagen®) was followed for this process. All plasma eluates were stored in 1.5 mL DNA LoBind micro-centrifuge tubes (Eppendorf®). Additionally, all plasma cfDNA samples were lyophilized prior to mutation detection analyses. Lyophilization was performed using the VirTis BenchTop Pro Freeze Dryer with OmnitronicsTM (SP Scientific). Samples (40–70 μL) were flash-frozen in liquid nitrogen, lyophilized for approximately 3 h and reconstituted in 5–25 μL H_2_O for 2 h at 4 °C before testing.

### Animal study

All in vivo studies with *Lgr5Cre*^*ER*^*-Apc*^*fl/fl*^ mice were carried out under the project licence (PPL) no. 60/4370 granted by the Home Office. Animal studies were conducted at the Preclinical Research Facility, University of Leicester, UK. Mice were housed in a climate-controlled environment with a 12 h day/night cycle and fed EURodent Diet 14% (5LF2*). Intraperitoneal injection of tamoxifen (10 mg/mL tamoxifen in sunflower oil) was used to induce loss of *Apc* in mice. The vehicle control was prepared by mixing sunflower seed oil and absolute ethanol in a 9:1 ratio at room temperature. Mice were injected with 100 μL/10 g of body weight. All the study mice were monitored by trained staff at the Preclinical Research Facility, University of Leicester, UK. Mice with *Lgr5Cre*^*ER*^*-Apc*^*fl/fl*^ were allocated randomly to tamoxifen injection or vehicle control. Sample size was not determined by power analysis.

Small intestine and colon were harvested and fixed in 10% formalin overnight before being processed for paraffin embedment by Core Biotechnological Services, University of Leicester, UK. Small intestine was divided into 3 or 6 sections and Swiss-rolled for embedding. Small pieces of tissue (approximately 3 × 3 mm) were also collected from each tissue section and stored at −80 °C for DNA extraction. Haematoxylin and eosin (H&E) staining of FFPE samples was performed to examine any pathological changes in the tissues. H&E staining was performed by Core Biotechnological Services at University of Leicester, UK. Histological images were acquired with NanoZoomer-XR digital slide scanner.

### DNA extraction from murine tissue, stool and blood plasma

Intestinal tissue DNA was extracted using the DNeasy® Blood & Tissue Kit (Qiagen®). Frozen tissues stored at either −20 °C or −80 °C were left to equilibrate to room temperature, and were cut into small pieces (maximum weight = 25 mg). Then, 180 μL buffer ATL and 20 μL Proteinase K were added and samples were digested at 56 °C overnight. Next, samples were left to cool and 4 μL 100 mg/mL RNase A was added. Following this, 400 μL each of buffer AL and 100% ethanol were added. Samples were loaded onto the DNeasy membrane columns. Columns were washed with 500 μL Buffer AW1 by centrifugation at 6000 × *g* for 1 min and 500 μL of Buffer AW2 by centrifugation at 20,000 × *g* for 3 min. DNA was eluted with 70 μL Buffer AE by centrifugation at 6000 × *g* for 1 min.

Blood was collected in 1.3 mL EDTA-coated tubes (K3E, TEKLAB™) by cardiac puncture under terminal anesthesia with isofluorane. Tubes were kept on ice and plasma was isolated within 2 h by sequential centrifugation at 1000 × *g* for 10 min and then at 2000 × *g* for 15 min to remove residual cellular components. Plasma volumes ranged from 150 to 900 μL and samples were stored at −80 °C until use. QIAmp® DNA Blood Mini Kit (Qiagen®) was used for DNA isolation. For plasma samples below 200 μL, phosphate-buffered saline was added to reach 200 μL minimum volume. Proteins were digested by adding Qiagen protease (10 μL per 100 μL plasma). Buffer AL was added in a 1:1 ratio and incubated at 56 °C for 20 min. After incubation, 100% ethanol was added in a 1:1 ratio and the resulting solution was passed through the QIAmp silica-gel membrane columns by centrifugation at 6000 × *g* for 1 min. Column washing and eluting steps were as described above.

Feces were collected on a weekly basis. For collection, each mouse was transferred to a sterile cage, monitored until excretion of fecal material and the collected sample immediately snap-frozen in liquid nitrogen and stored at −80 °C. Quantities of fecal samples collected ranged from 9 to 234 mg (median = 39 mg). DNA was extracted using the QIAamp® Fast DNA Stool Mini Kit (Qiagen®) according to the manufacturer’s instruction. Eluted DNA samples were stored at −20 °C.

### Multiregional targeted NGS of patient adenoma samples

NGS analysis for FFPE samples (*n*=30) from 6 patient cases (H149, H154, H263, H264, H265 and H266) was performed at the Wellcome Sanger Institute, Cambridge, UK, using a customized targeted CRC panel (SureSelect, Agilent, UK) consisting of all coding exons of 116 genes, 22 genes recurrently amplified/deleted, 51 copy number regions, 121 MSI regions and 2 gene fusions (RSPO2 and 3). Samples were fragmented to an average insert size of 150 bp and subjected to Illumina DNA sequencing library preparation using Bravo automated liquid handling platform. Sequencing was performed on an Illumina HiSeq2000 machine using the 75 bp paired-end protocol with the target of 1 Gb sequence per sample. Data quality was checked for 95% target coverage at 100× and mutation analysis was performed using an in-house algorithm. Sequencing reads are aligned to the National Center for Biotechnology Information (NCBI) build 37 human genome using the BWA algorithm with Smith–Waterman correction with PCR duplicates removed^56^. Base substitutions, small insertions or deletions and breakpoints were identified by comparison against an unmatched control using established bioinformatic algorithms: CaVEMan for mutations^57^; Pindel to detect insertions and deletions^58^; and CNVKit for copy number detection (https://github.com/etal/cnvkit). The control used is an unmatched blood sample sequenced to an equivalent depth. To account for the absence of matched control, a bespoke variant selection pipeline was developed. To enrich for high-confidence somatic variants, there was further filtering by removing: known constitutional polymorphisms using human variation databases: Ensembl GRCh37.5, 1000 genomes release 2.2.2 and ESP6500; and alterations where the same sequence change was present at least twice in exome or whole-genome sequencing data derived from 317 constitutional (normal) DNA samples.

### Primers and probes

The Primer Express (v3.0) software (Applied Biosystems™) was used to design custom primers and probes for various assays. Primers were designed to be 18–26 bp in length, 40–60% GC content and within the melting temperature (*T*_m_) range of 58–62 °C. The length of the amplicon was limited to <100 base pairs. Tentative primer pairs were then analyzed with the University of California, Santa Cruz (UCSC) in silico PCR platform (https://genome.ucsc.edu/cgi-bin/hgPcr). Primer pairs were validated using SYBR Green Real-Time PCR. To ensure that probes annealed to their target alleles before primer-target hybridization occurred, TaqMan™ Minor-Binding-Groove (MGB) probes were designed to be approximately 10 °C higher in *T*_m_ compared to their primer counterparts. Probe were designed to discriminate WT and mutant sequences, other parameters included 15–20 bp in length, 40–60% GC content, and the last 5 nucleotides at 3’ to contain no more than 2 G/C nucleotides to avoid the “GC clamping” effect. For mutation-specific probes, the mutant bases were placed in the middle of the amplicon for optimal mismatch discrimination^59^. Peptide nucleic acids (PNAs; Eurogentec, Belgium) were designed as 13–15b oligomers with the *T*_m_ of ~70 °C (~10 °C higher than *T*_m_ of primers) and sequences complimentary to WT alleles.

### DNA quantification of patient plasma samples

The quantification analysis was run on the StepOnePlus^TM^ Real-Time PCR System Thermal Cycling Block (ABI^®^). Plasma DNA samples were quantified using an intronic sequence of the housekeeping human *GAPDH* gene. Then, 10 ng of human genomic DNA (hgDNA) control (Roche, Germany) was diluted 1:2 ratio in H_2_O for 7–8 dilutions in triplicate to construct a standard curve. The assay was run in a 10 μL reaction volume containing 5 μL 2× TaqMan® Genotyping PCR Master Mix, 600 nM primers, 100 nM of the TaqMan probe and 3 μL of DNA sample. The PCR program was 50 °C for 2 min, 95 °C for 10 min and 50 cycles of 95 °C for 15 s followed by 60 °C for 1 min at ramp rate of 1.6 °C/s. Data analysis was done on the StepOnePlus^TM^ Software v2.3 (ABI^®^). Primers and probe sequences are reported in Supplemental Table 5.

### DNA quantification of patient FFPE samples

Patient FFPE tissue DNA samples were quantified using an assay designed to amplify a 69 bp targeting the *Alu* retrotransposon elements^60^. Next, 10 ng of human genomic DNA (Roche) was diluted 1:2 ratio in H_2_O for 7–8 dilutions to construct a standard curve. Each dilution was run in triplicate. Reactions were run in 10 μL volumes containing 5 μL 2× TaqMan® Genotyping PCR Master Mix, 600 nM of each primer, 100 nM probe and 3 μL of DNA sample. The assay was run using the programme: 50 °C for 2 min, 95 °C for 10 min and 50 cycles of 95 °C for 15 s followed by 60 °C for 1 min.

### *BRAF* and *KRAS* mutation analysis on FFPE samples using real-time PCR

The three assays *BRAF-c.1799*, *KRAS-c.34* and *KRAS-c.35* were run on StepOnePlus^TM^ Real-Time PCR (ABI^®^). The mutant probe for the *KRAS-c.34* and *KRAS-c.35* assays incorporate a degenerate D (A/T/G) nucleotide for the detection of three possible missense point mutations at each hotspot. To enhance detection of the mutant DNA, WT gene signal was inhibited using peptide nucleic acid (PNA)^61^: 150 nM of PNA per reaction well for the *BRAF-c.1799* assay and 60 nM of PNA for the *KRAS-c.34* and *KRAS-c.35* assays. The *BRAF-c.1799* and *KRAS-c.35* assays were run in 10 μL reaction volumes containing 5 μL of 2× TaqMan® Genotyping PCR Master Mix, 600 nM primers and 100 nM of WT and mutant probes. Each reaction contained 3 μL of sample and each sample was run in triplicate. The PCR program was: holding stage at 50 °C for 2 min followed by 95 °C at 10 min, then 50 cycles of 95 °C for 15 s and 60 °C for 1 min at ramp rate of 1.6 °C/s. Data analysis was done on the StepOnePlus^TM^ Software v2.3 (ABI^®^). Primer, probe and PNA sequences are reported in Supplemental Table 5.

### Mutation detection assays using the ddPCR system

All ddPCR assays were run on the QX200^TM^ ddPCR system (BioRad). Each sample was run in 20 μL volume containing 10 μL ddPCR Supermix for Probes (BioRad). Within 1 h of droplet generation, reaction plates were run on the C1000 Touch^TM^ Thermal Cycler with 96-Deep Well Reaction Module at a ramp rate of 2 °C/s. The FFPE DNA samples were checked for point mutations using sequence-specific TaqMan probes. For all assays, except *KRAS-c.38*, each sample was tested in 20 μL reaction volumes containing respective 450 nM of each primer, and WT and mutant probe at 250 nM. The *KRAS-c.38* assay was run in a 20 μL reaction volume containing 10 μL ddPCR Supermix for Probes, 1 μL each of *KRAS-c.35* WT and mutant mixes (dHsaCP2000013-14; BioRad^®^). The three mutation assays, *BRAF-c.1799*, *KRAS-c.34* and *KRAS-c.35*, were run on the following program: enzyme activation at 95 °C for 10 min, 40 cycles of denaturation and extension at 94 °C for 30 s and 59 °C for 1 min, respectively, enzyme deactivation at 98 °C for 10 min with holding at 4 °C. The *KRAS-c.38* assay was also run on the same PCR program except at 55 °C for 1 min during the extension stage. The *APC-c.4012*, *APC-c.4189*, *TP53-c.817* and *PI3K-c.3140* assays were run using the same PCR program except at 57 °C for 1 min during the extension stage. As for the *APC-c.4285* assay, the extension temperature was increased to 60 °C for 1 min. Primers and probe sequences are reported in Supplemental Table 1. Droplets were read using the QX200^TM^ Droplet Reader and data analyzed with QuantaSoft^TM^ software (version 1.6.6.0320). Threshold calling was set at the limit of detection (LoD), which is defined by the minimum target concentration that can be discriminated from the non-target (WT) background. Assuming a false-positive rate of 0 droplet and a LoD of 0.01% for single-well assays, at least 3 mutant copies need to be detected above the fluorescence threshold in each well for positive target (mutant) calling at 95% statistical confidence interval. cfDNA-like positive control containing *BRAF-T1799A* and *KRAS-G35* mutations was purchased from SeraCare and 10 ng was run on the ddPCR platform.

### DNA quantification of murine samples

To quantify murine DNA samples, we implemented a ddPCR assay (BioRad QX200) for amplification of mouse *Gapdh* pseudogenes^42^. To construct a standard curve, 10 ng of mouse genomic DNA control (Promega) was diluted in 1:2 ratios in H_2_O for 7–8 dilutions. Each dilution was run in either duplicate or triplicate. Reactions were run in 10 μL volume containing 5 μL 2× TaqMan® Genotyping PCR Master Mix, 600 nM of each primer, 100 nM probe and 3 μL of sample. The qPCR was run using the following program: 50 °C for 2 min, 95 °C for 10 min and 50 cycles of 95 °C for 15 s followed by 60 °C for 1 min. Primer and probe sequences are reported in Supplemental Table 6.

### Analysis of recombined alleles in *Lgr5Cre*^*ER*^*-Apc*^*fl/fl*^ mice

Reactions were run on BioRad QX200 digital PCR platform in a 10 μL volume containing 5 μL 2× TaqMan® Genotyping PCR Master Mix, 600 nM of each primer, 100 nM of probe and 3 μL of sample. The assay was run using the following program: 50 °C for 2 min, 95 °C for 10 min and 50 cycles of 95 °C for 15 s followed by 60 °C for 1 min. Primers and probe sequences are reported in Supplemental Table 6.

### Statistical analysis

Total plasma cfDNA concentrations between the control and experimental groups were analyzed using Student's *t*-test or non-parametric Mann-Whitney test. Efficiencies of real-time PCR and ddPCR assays were calculated using linear regression analysis. For all mutation detection assays performed on ddPCR, Poisson distribution analysis was used to predict the probability of independent and random distribution of targets into droplets at 95% confidence level. Data were analyzed using GraphPad Prism 7 or Excel software packages, unless otherwise stated.

## Electronic supplementary material

Supplementary data

Supplementary Table 1

Supplementary Table 2

Supplementary Table 3

Supplementary Table 4

Supplementary Table 5

Supplementary Table 6

Supplementary figure legends
