# Supplementary material for: Circulating tumor DNA in patients with colorectal adenomas: assessment of detectability and genetic heterogeneity
Source: Cell Death Dis. 2018 Aug 30;9(9):894. doi: 10.1038/s41419-018-0934-x (PMC6117318; doi:10.1038/s41419-018-0934-x)
Supplement: Supplementary file 1 — Supplementary data [file 41419_2018_934_MOESM1_ESM.pdf]

# Supplemental methodology

## Identification of loxP sites in the *Lgr5Cre$^{ER}$-Apc$^{fl/fl}$* mice

The floxed *Apc* allele used for the generation of the *Lgr5-EGFP-IRES-cre$^{ERT2+/0}$;Apc$^{fl/fl}$* (*Lgr5Cre$^{ER}$-Apc$^{fl/fl}$*) was originally developed by Shibata and colleagues[42]. Two 34bp *loxP* (**L**ocus **of** **X**(cross)-over in **P**1) sites were inserted in introns 13 and 14, thus flanking exon 14 of the endogenous *Apc* locus. The second *LoxP* site in intron 14 is preceded by a Neomycin selection cassette, as shown below.

To design primers and probes for custom PCR assays for *in vivo* analysis, we

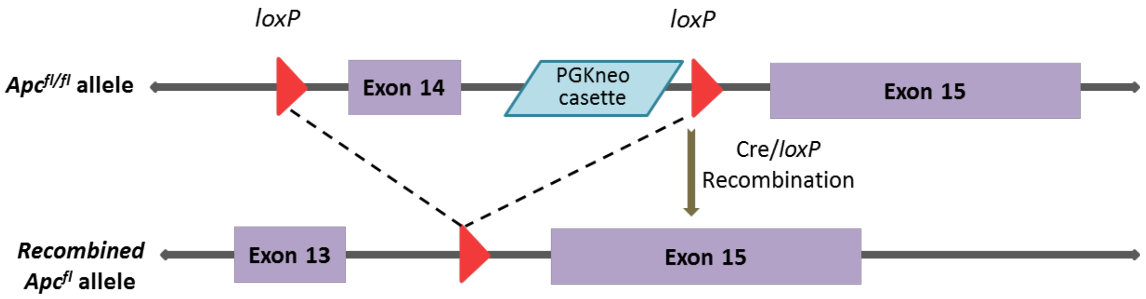


**Schematic of the *Apc$^{fl/fl}$* Allele.** The picture shows the location of the 2 *loxP* sites and the neomycin selection cassette.

sequenced the genomic regions encompassing the two *loxP* sites in the *Apc$^{fl/fl}$* allele. We designed a PCR assay (see below) to amplify 308bp containing the upstream *loxP* region within intron 13 of the *Apc$^{fl/fl}$* allele using genomic DNA extracted from ear biopsy tissues of a genotyped *Lgr5Cre-Apc$^{fl/fl}$* mice. DNA was purified after PCR amplification and used for Sanger sequencing with both forward and reverse primers. No discordances in the sequences were observed in 3 separate animals. Additionally, the endogenous mouse sequences surrounding the *loxP* were confirmed using the BLAST programme (blastn suite). The resulting sequence is reported below.

.

5’………GTTCTGTATCATGGAAAGATAGGTGGTCATTAGTTTAATCCTGTGTTGAT
CCT**ATAACTTCTTATAGCATACATTATACGAAGTTAT**CGAGCTTGACCACCAAA
CCCGGGCTTTGCTGACGAATTCGGAGACTCATAGAAACAGCACTGACCCAAA
TTTCATTTTGTGTGAAACTGTAAATGAAAGGTTCCTGATTTACTAGTGAGGAAT
GTCAGAAGGGAGACCAAAAGAAAAAAGACTCTTAACTAAATGGCACATACTG
TGTTTCAATCGTGACTAGGAATCAACCCTCAAAAGCGTTTTGAGTG…………… -3’

**Sequence details for the Upstream *loxP* site in the *Apc*$^{fl/fl}$ Allele.** Amplified 308bp bands for three distinct mouse samples. Positive (+ve) control was the DNA sample from an *Lgr5Cre*$^{ER}$*-Apc*$^{fl/fl}$ mouse. Negative (-ve) control was water. TrackIt™ 100bp DNA ladder was used a size marker. **C)** The sequencing data for the upstream *loxP* region. The mismatch thymidine is in red. *Blue* = intron 13, *green* = exon 14, *yellow* = *Hinc II* restriction sites, *underlined* = upstream *loxP* region.

Interestingly, the sequenced *loxP* site showed a guanosine (G) to thymidine (T) change compared to the consensus reference sequence:

5’ ATAACTTC**T**TATAGCATACATTATACGAAGTTAT 3’ (sequenced *loxP*)
5’ ATAACTTC**G**TATAGCATACATTATACGAAGTTAT 3’ (published *loxP*)

The reason for this discrepancy is not known. However, successful *loxP* recombination was observed in tamoxifen-treated *Lgr5Cre*$^{ER}$*-Apc*$^{fl/fl}$ mice.

As mentioned, intron 14 contains a phosphoglycerate kinase 1 (PGK1) promoter and a neomycin (neo) resistance gene inserted upstream of the *loxP* sequence. To sequence over 1,000bp, a semi-nested PCR approach was adopted. The first PCR was run using the PGK-F forward primer and the P5-2 reverse primer to produce a 1,596bp amplicon (see below for methodological details). Then, the Sanger sequence obtained from this initial amplicon was used to design a second primer pair (P5-3F and P5-3R, see below) to amplify and sequence a shorter internal amplicon of 736bp to complete the whole sequence shown below.

5'............**CATTCTGCACGCTTCAAAAG**CGCACGTCTGCCGCGCTGTTCTCCTC
TTCCTCATCTCCGGGCCTTTCGAC**CTGCAG**CCAATATGGGATCGGCCATTGAA
CAAGATGGATTGCACGCAGGTTCTCCGGCCGCTTGGGTGGAGAGGCTATTCG
GCTATGACTGGGCACAACAGACAATCGGCTGCTCTGATGCCGCCGTGTTCCGG
CTGTCAGCGCAGGGGCGCCCGGTTCTTTTTGTCAAGACCGACCTGTCCGGTG
CCCTGAATGAA**CTGCAG**GACGAGGCAGCGCGGCTATCGTGGCTGGCCACGAC
GGGCGTTCCTTGCGCAGCTGTGCTCGACGTTGTCACTGAAGCGGGAAGGGAC
TGGCTGCTATTGGGCGAAGTGCCGGGGCAGGATCTCCTGTCATCTCACCTTGC
TCCTGCCGAGAAAGTATCCATCATGGCTGATGCAATGCGGCGGCTGCATACGC
TTGATCCGGCTACCTGCCCATTCGACCACCAAGCGAAACATCGCATCGAGCGA
GCACGTACTCGGATGGAAGCCGGTCTTGTCGATCAGGATGATCTGGACGAAG
AGCATCAGGGGCTCGCGCCAGCCGAACTGTTCGCCAGGCTCAAGGCGCGCAT
GCCCGACGGCGATGATCTCGTCGTGACCCATGGCGATGCCTGCTTGCCGAATA
TCATGGTGGAAAATGGCCGCTTTTCTGGATTCATCGACTGTGGCCGGCTGGGT
GTGGCGGACCGCTATCAGGACATAGCGTTGGCTACCCGTGATATTGCTGAAGA
GCTTGGCGGCGAATGGGCTGACCGCTTCCTCGTGCTTTACGGTATCGCCGCTC
CCGATTCGCAGCGCATCGCCTTCTA**TCGCCTTCTTGACGAGTTCT**TCTGAGGG
GATCCGCTGTAAGTCTGCAGAAATTGATGATCTATTAAACAATAAAGATGTCCA
CTAAAATGGAAGTTTTTCCTGTCATACTTTGTTAAGAAGGGTGAGAACAGAGT
ACCTACATTTTGAATGGAAGGATTGGAGCTACGGGGGTGGGGGTGGGGTGG
GATTAGATAAATGCCTGCTCTTTACTGAAGGCTCTTTACTATTGCTTTATGATAA
TGTTTCATAGTTGGATATCATAATTTAAACAAGCAAAACCAAATTAAGGGCCAG
CTCATTCCTCCCACTCATGATCTATAGATCTATAGATCTCTCGTGGGATCATTGT
TTTTCTCTTGATTCCCACTTTGTGGTTCTAAGTACTGTGGTTTCCAAATGTGTC
AGTTTCATAGCCTGAAGAACGAGATCAGCAGCCTCTGTTCCACATACACTTCA
TTCTCAGTATTGTTTTGCCAAGTTCTAATTCCATCAGAAGCTGGTCGATCGAAT
TCCTGCAGCCCGGGGGATCCT**ATAACTTCTTATAGCATACATTATACGAAGTTA**
**T**CGGATCCACTAGTTCTAGCATTATATTGACTGTTAGCCCTTATATTTATATGCTT
TTTGTATTTTTAAAACCTATACTTCATGTTATTTCTTAAAATAATGCTTATATACA
CAGTCTGCCAAAGTGTGCTTTGGACTTGGTGTCTTCACT***GAGACAGAGACCC***
***CGTACTC***.........3'

**Downstream *loxP* Sequence in Intron 14 of *Apc*$^{fl/fl}$ Allele.** As with the upstream *loxP* sequence, a base change of G>T (in red) was observed at the same location in the palindromic region of *loxP*. The underlined nucleotide 'C' at the start of intron 14 (green) represents the 5'-sticky end of the *Sac I* digestion site where 2nd *loxP* sequence was inserted. *Orange* = PGK-F primer, *grey* = 2691-3582bp region in PGKneobpA plasmid, *yellow* = *PstI* restriction sites, *pink* = P5-3F primer, *blue* = partial alignment with plasmid vector β-lactamase gene (pHM2/3), *underlined* = *loxP*, *green* = intron 14, *purple* = P5-3R primer.

Using the PCR-amplified products from the two PCR reactions, we obtained a 1,596bp sequence of the downstream *loxP* and surrounding regions. The sequencing data was confirmed using BLAST (blastn suite: Align) to position the *loxP* site respective to the intronic and exonic regions of the endogenous *Apc* gene. The first 892 bases of sequenced data showed alignment with 2,691-3,582 bp region of the PGKneobpA plasmid (grey), and contained the *Pst1* restriction sites (yellow)[42]. The following 467 bases (blue) showed partial alignment with plasmid vector β-lactamase gene (pHM2/3), likely a residual gene region from the original construct using for homologous recombination[42]. The *loxP* was inserted at the sticky end 'C' (5'-GAGCTC-3'; underlined & green) produced by *Sac I* digestion at the start of intron 14 (green). Alignment with the intron 14 of *Apc* was confirmed using the GRCm38.p4 assembly of C57BL/6J strain (blastn suite). Interestingly, like the 1st *loxP* site, the 2nd *loxP* sequence also presented a G>T base change (in red) from the consensus *loxP* site sequence. Both upstream and downstream *loxP* sites were accompanied by unique sequences that were probably derived from the original DNA construct used for somatic recombination.

Using the sequencing data, custom primers and probes were designed for the detection of recombined and non-recombined *Apc*$^{fl/fl}$ alleles. Cre-mediated recombination leads to deletion of *Apc* exon 14 bringing the introns 13 and 14 together. The customized assay for detection of the recombined *Apc* allele was designed to amplify a 102bp region encompassing the recombined loxP sites (Supplemental Figure 9). The Apcfl-102NF1 forward primer was designed to partially anneal with the unique sequence upstream of the *loxP* site and intron 13 to improve target specificity, and the Apcfl-102NR2 reverse primer annealed to the intron 14 sequence adjacent to the recombined *loxP*. A TaqMan probe (Apcfl-P1) tagged with a FAM reporter dye was finally targeted to the palindromic region of the *loxP* site.

The detection of the non-recombined *Apc*$^{fl/fl}$ allele was achieved by an assay tailored to a 75bp region within the PGKneo cassette that is only present in the non-recombined alleles, as this region is excised out during Cre-mediated recombination (Supplemental Figure 9). The use of a sequence-specific TaqMan probe (Apcfl-NRP1) tagged with VIC reporter dye enabled the both assays to be run in parallel. By doing so, the fractional abundance of recombined *Apc*$^{fl/fl}$ alleles could be calculated using the background non-recombined alleles as a reference.

**DNA Extraction from Murine Ear Tissues**

Ear biopsies were processed using Gentra kit. Tissue was lysed in 300µL of cell lysis solution by vortexing, and then 1.5µL of Proteinase K solution and incubated at 55ºC overnight. Following this, 100µL of protein precipitation solution was added followed by centrifugation at 13,000rpm for 3 minutes. Supernatant was recovered and 300µL 100% Isopropanol was added before centrifugation at 13,000rpm for 3 minutes. DNA pellet was washed with 300µL 70% ethanol, air-dried and re-suspended 1 hour at 65ºC in 50µL DNA Hydration solution.

**PCR for *loxP* regions of the *Apc$^{fl/fl}$* Allele**

PCR reaction contained 5µL 10xPCR Run Buffer, 2.5mM MgCl2, 20µM dNTP Mix, 0.2µL Taq DNA Polymerase, 400nM of each primer and $H_2O$ to 50µL. 0.5µL DMSO per reaction were used to amplify the downstream Apc$^{fl/fl}$ locus. Reagent were from Invitrogen. Each reaction was run with 2.5µL of DNA extracted from ear tissues on the GeneAmp® PCR System 9700 machine, using the following programmes:

- Upstream floxed region: 95ºC for 3 minutes, 40 cycles of 95ºC for 30 seconds, 60ºC for 30 seconds, 72ºC for 1 minute, and a final 72ºC step for 5 minutes;
- Downstream floxed region: 95ºC for 3 minutes, 40 cycles of 95ºC for 30 seconds, 58ºC for 45 seconds, 72ºC for 150 seconds, and a final 72ºC step for 10 minutes.

The primers used are shown below.

| *Name* | *Type* | *Sequence (5'-3')* | *Amplicon size (bp)* | *Assay* | *Target* |
|---|---|---|---|---|---|
| Apc-P3 | FP | GTTCTGTATCATGGAAAGATAGGTGGTC | 308 (*Apc$^{fl/fl}$*); 226 (*Apc$^{WT}$*) | Apc-lox1 | Upstream (1st) *loxP* region in the *Apc$^{fl/fl}$* allele |
| Apc-P4 | RP | CACTCAAAACGCTTTTGAGGGTTG | | | |
| PGK-F | FP | CATTCTGCACGCTTCAAAAG | 1,596 | Apc-lox2 | Downstream (2nd) *loxP* region in the *Apc$^{fl/fl}$* allele |
| P5-2 | RP | GAGTACGGGGTCTCTGTCTCAG | | | |
| P5-3F | FP | TCGCCTTCTTGACGAGTTCT | 736 | | |
| P5-3R | RP | GAGTACGGGGTCTCTGTCTC | | | |

## Gel Electrophoresis

The PCR products were analysed by gel electrophoresis in 1-2% Agarose gel in TBE buffer. 5μL Ethidium Bromide was added per 100mL gel for DNA visualisation under UV light. 2μL Gel Loading Dye was mixed with 10μL sample. The gel was run in TBE buffer at 100V for 40 minutes using Labnet ENDURO™ GEL XL machine. 5μL TrackIt™ 100bp DNA Ladder was used as the DNA size marker and the gel was visualised using the SynGene™ UV Transilluminator and the GeneSnap™ software (version 7.12).

## Purification & Sequencing of PCR products

PCR DNA products were purified using the DNA Clean & Concentrator™-5 kit (Zymo Research). A pipette tip was used to prick the bands in the gel to collect the DNA products. The tip was swirled in 10μL d$H_2O$ to recover the DNA and 50μL DNA binding buffer was added. The mixture was passed through the Zymo-Spin™ column by centrifugation at 8,000rpm for 1 minute. The columns were washed twice with 200μL DNA wash buffer by centrifugation at 8,000rpm for 1 minute. The column matrix was incubated in 50μL DNA elution buffer at room temperature for 5 minutes. DNA was eluted by centrifugation at 8,000rpm for 1 minute. DNA aliquots were quantified using NanoDrop™ Spectrophotometer (ND-1000). Sequencing of the PCR products was carried out by the PNACL (Protein Nucleic Acid Chemistry Laboratory, The Centre for Core Biotechnology Services, University of Leicester, UK) facility using an automated Applied Biosystems™ 3730 Sanger sequencer.

A)

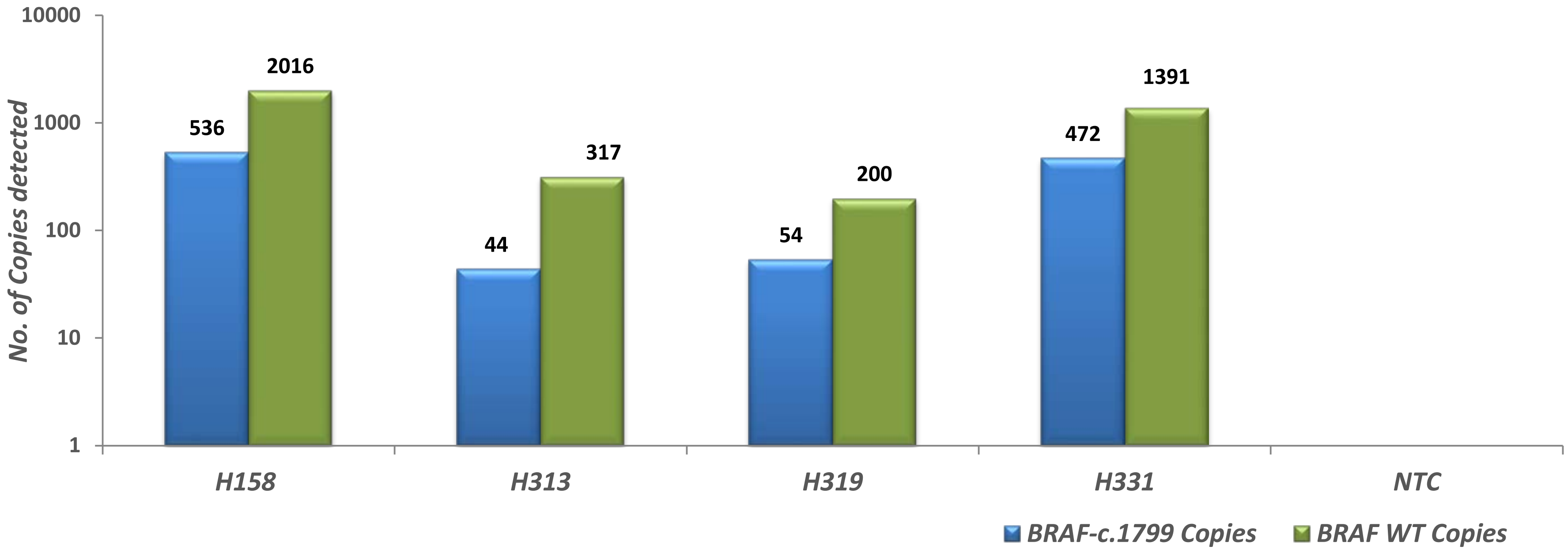


B)

| Sampl e ID | % Mutant Fractional Abundance in FFPE samples | Histology | Polyp Dimension s (mm) |
|---|---|---|---|
| H158 | 26.6 | Sigmoid (benign HP) | 7 x 5 x 4 |
| H313 | 13.9 | Rectal (HP) | 4 |
| H319 | 27 | Rectal (HP) | 5 x 4x 3 |
| H331 | 33.9 | Proximal Sigmoid (LG VA) | <10 |

*Moe Myint et al_Supplemental Figure 1*

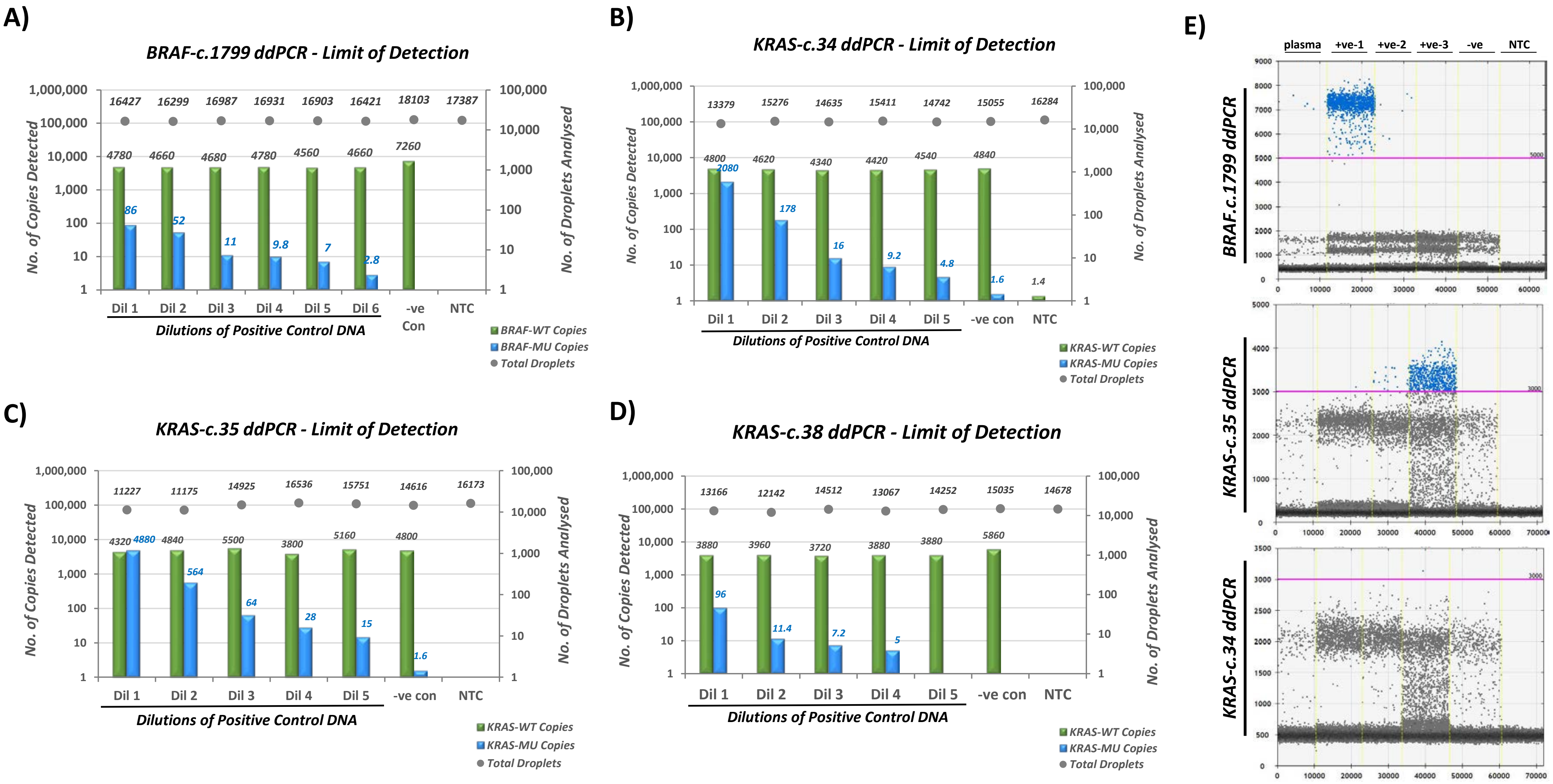


Moe Myint et al_Supplemental Figure 2

A)

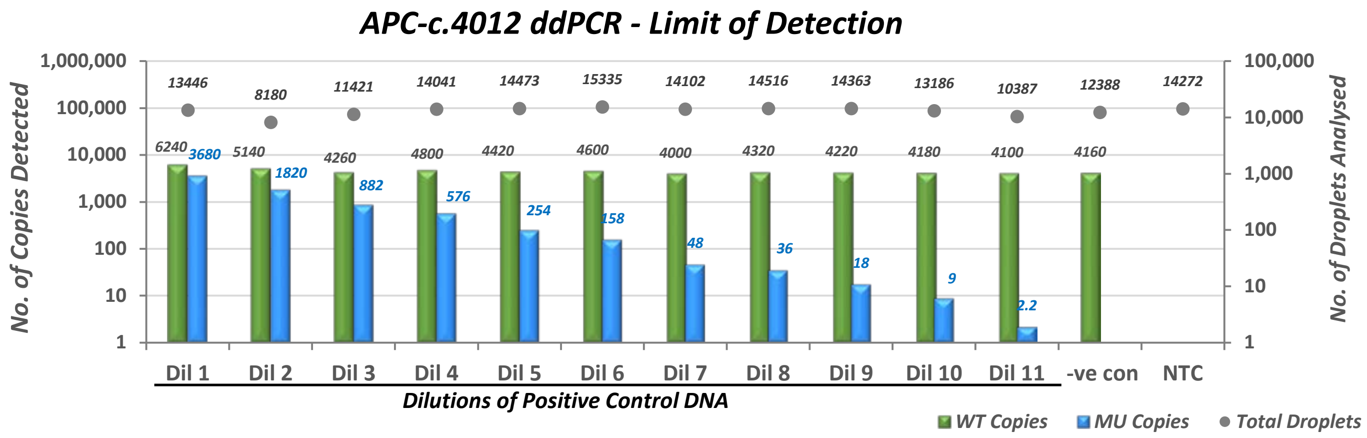


B)

| Dilution | Ratio to Background DNA | Pos Con Quantity (pg) | Mutant Copies Detected | Poisson Max Mutant Copies | Poisson Min Mutant Copies |
|---|---|---|---|---|---|
| 1 | 1:1 | 5000 | 3680 | 3840 | 3520 |
| 2 | 1:3 | 2500 | 1820 | 1960 | 1680 |
| 3 | 1:7 | 1250 | 882 | 966 | 798 |
| 4 | 1:15 | 625 | 576 | 636 | 514 |
| 5 | 1:31 | 312.5 | 254 | 296 | 216 |
| 6 | 1:63 | 156.25 | 158 | 190 | 128 |
| 7 | 1:127 | 78.13 | 48 | 68 | 32 |
| 8 | 1:255 | 39.06 | 36 | 52 | 22 |
| 9 | 1:511 | 19.53 | 18 | 32 | 10 |
| 10 | 1:1023 | 9.77 | 9 | 19.4 | 3.2 |
| 11 | 1:2047 | 4.88 | 2.2 | 10.8 | 0.0 |
| -ve con | N/A | 0 | 0 | 0 | 0 |
| NTC | N/A | 0 | 0 | 0 | 0 |

C)

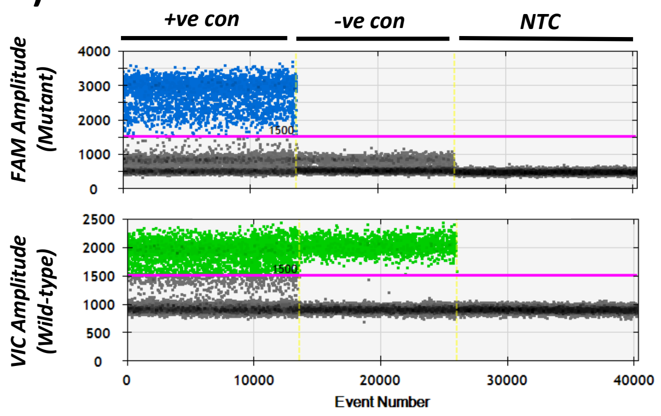


D)

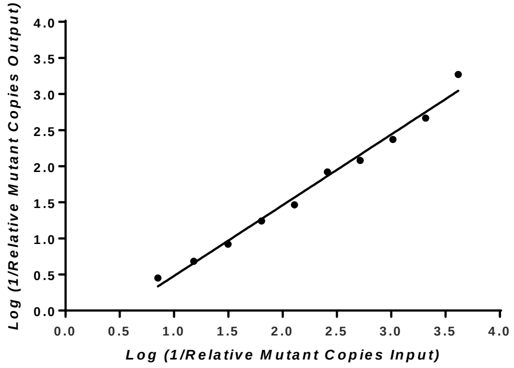


Moe Myint et al_Supplemental Figure 3

A)

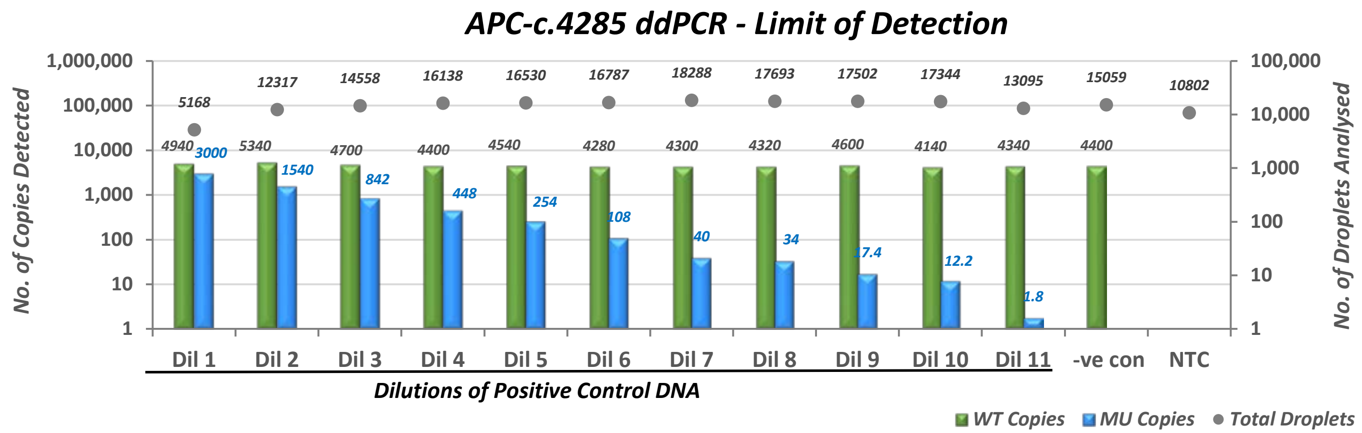


B)

| Dilution | Ratio to Background DNA | Pos Con Quantity (pg) | Mutant Copies Detected | Poisson Max Mutant Copies | Poisson Min Mutant Copies |
|---|---|---|---|---|---|
| 1 | 1:1 | 5000 | 3000 | 3240 | 2760 |
| 2 | 1:3 | 2500 | 1540 | 1640 | 1420 |
| 3 | 1:7 | 1250 | 842 | 916 | 770 |
| 4 | 1:15 | 625 | 448 | 500 | 398 |
| 5 | 1:31 | 312.5 | 254 | 292 | 218 |
| 6 | 1:63 | 156.25 | 108 | 134 | 86 |
| 7 | 1:127 | 78.13 | 40 | 56 | 28 |
| 8 | 1:255 | 39.06 | 34 | 50 | 22 |
| 9 | 1:511 | 19.53 | 17.4 | 28.8 | 9.6 |
| 10 | 1:1023 | 9.77 | 12.2 | 22.2 | 5.8 |
| 11 | 1:2047 | 4.88 | 1.8 | 8.6 | 0.0 |
| -ve con | N/A | 0 | 0 | 0 | 0 |
| NTC | N/A | 0 | 0 | 0 | 0 |

C)

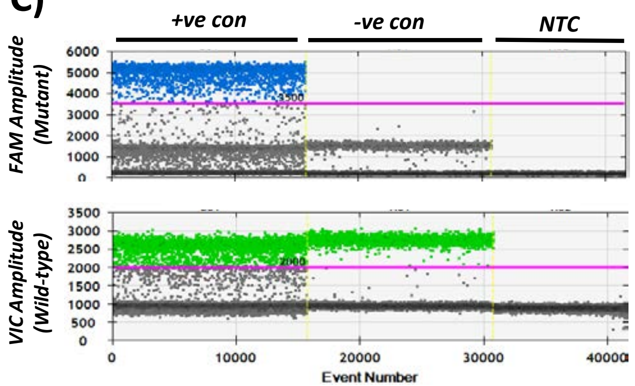


D)

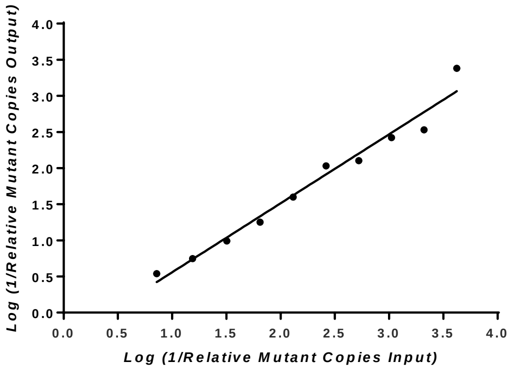


Moe Myint et al_Supplemental Figure 4

A)

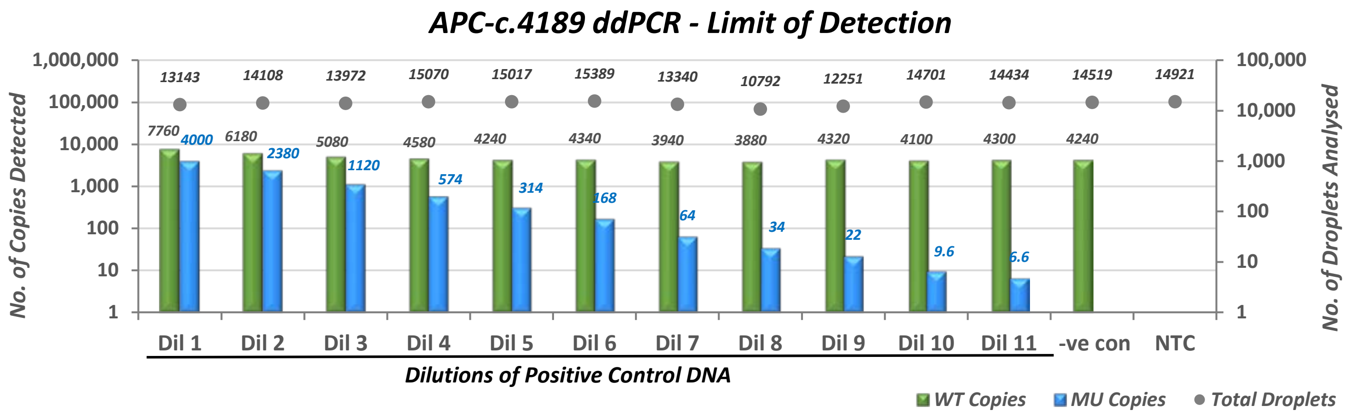


B)

| Dilution | Ratio to Background DNA | Pos Con Quantity (pg) | Mutant Copies Detected | Poisson Max Mutant Copies | Poisson Min Mutant Copies |
|---|---|---|---|---|---|
| 1 | 1:1 | 5000 | 4000 | 4180 | 3840 |
| 2 | 1:3 | 2500 | 2380 | 2500 | 2240 |
| 3 | 1:7 | 1250 | 1120 | 1206 | 1034 |
| 4 | 1:15 | 625 | 574 | 632 | 514 |
| 5 | 1:31 | 312.5 | 314 | 358 | 270 |
| 6 | 1:63 | 156.25 | 168 | 198 | 136 |
| 7 | 1:127 | 78.13 | 64 | 86 | 44 |
| 8 | 1:255 | 39.06 | 34 | 56 | 20 |
| 9 | 1:511 | 19.53 | 22 | 36 | 10 |
| 10 | 1:1023 | 9.77 | 9.6 | 19.6 | 3.8 |
| 11 | 1:2047 | 4.88 | 6.6 | 15.4 | 2.0 |
| -ve con | N/A | 0 | 0 | 0 | 0 |
| NTC | N/A | 0 | 0 | 0 | 0 |

C)

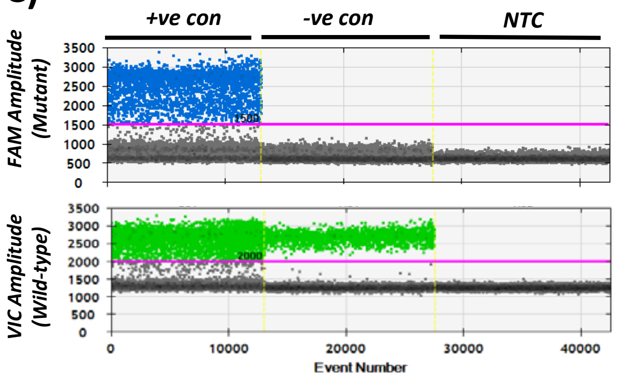


D)

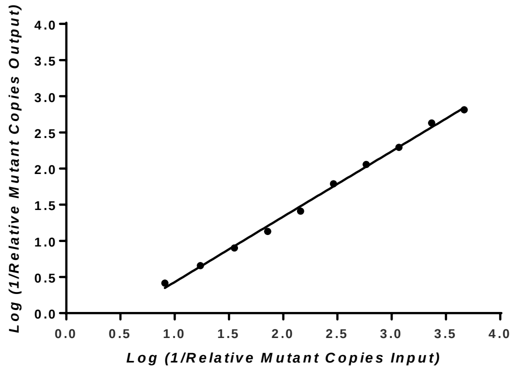


Moe Myint et al_Supplemental Figure 5

A)

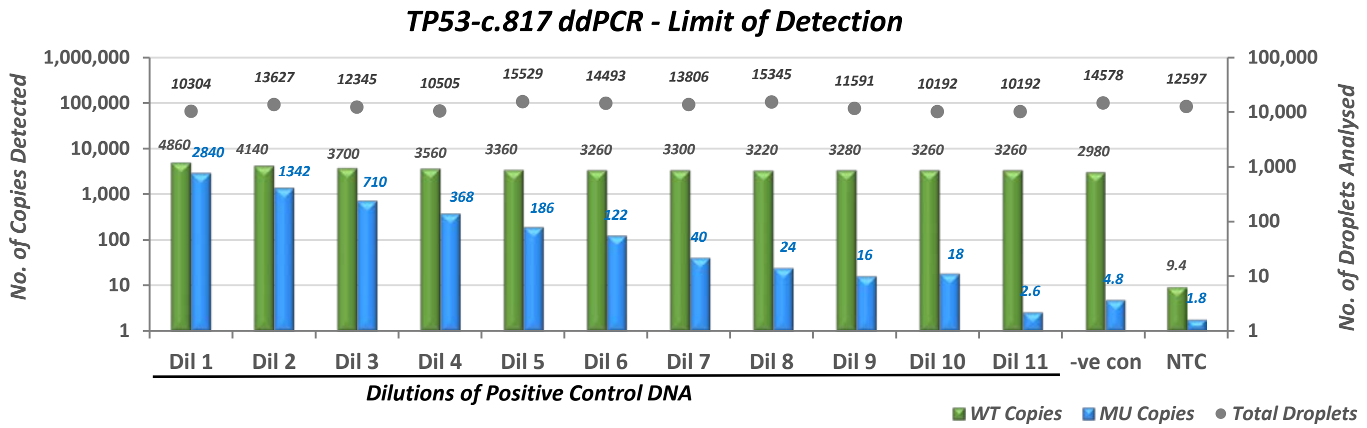


B)

| Dilution | Ratio to Background DNA | Pos Con Quantity (pg) | Mutant Copies Detected | Poisson Max Mutant Copies | Poisson Min Mutant Copies |
|---|---|---|---|---|---|
| 1 | 1:1 | 5000 | 2840 | 3020 | 2680 |
| 2 | 1:3 | 2500 | 1342 | 1438 | 1248 |
| 3 | 1:7 | 1250 | 710 | 782 | 638 |
| 4 | 1:15 | 625 | 368 | 424 | 312 |
| 5 | 1:31 | 312.5 | 186 | 218 | 152 |
| 6 | 1:63 | 156.25 | 122 | 152 | 96 |
| 7 | 1:127 | 78.13 | 40 | 58 | 26 |
| 8 | 1:255 | 39.06 | 24 | 38 | 14 |
| 9 | 1:511 | 19.53 | 16 | 30 | 8 |
| 10 | 1:1023 | 9.77 | 18 | 34 | 8 |
| 11 | 1:2047 | 4.88 | 2.6 | 12.2 | 0.2 |
| -ve con | N/A | 0 | 4.8 | 12.8 | 1.2 |
| NTC | N/A | 0 | 1.8 | 9 | 0 |

C)

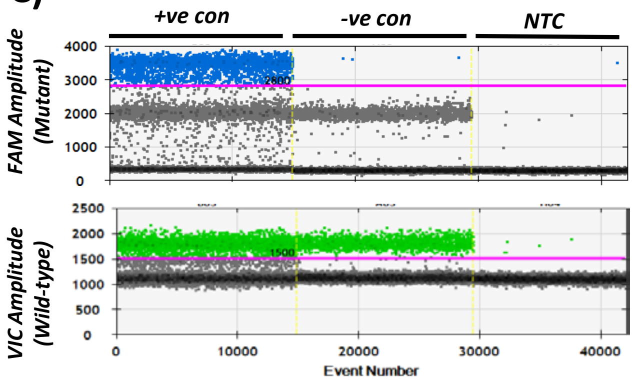


D)

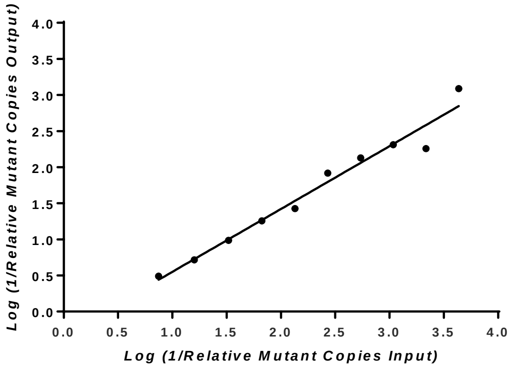


*Moe Myint et al_Supplemental Figure 6*

A)

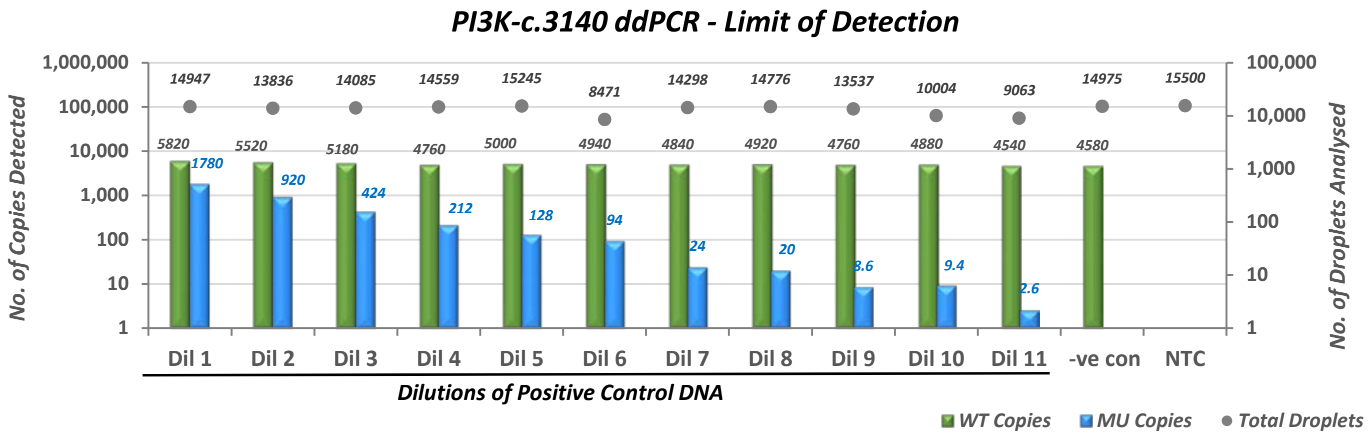


B)

| Dilution | Ratio to Background DNA | Pos Con Quantity (pg) | Mutant Copies Detected | Poisson Max Mutant Copies | Poisson Min Mutant Copies |
|---|---|---|---|---|---|
| 1 | 1:1 | 5000 | 1780 | 1880 | 1660 |
| 2 | 1:3 | 2500 | 920 | 998 | 840 |
| 3 | 1:7 | 1250 | 424 | 478 | 372 |
| 4 | 1:15 | 625 | 212 | 250 | 176 |
| 5 | 1:31 | 312.5 | 128 | 158 | 102 |
| 6 | 1:63 | 156.25 | 94 | 130 | 66 |
| 7 | 1:127 | 78.13 | 24 | 38 | 12 |
| 8 | 1:255 | 39.06 | 20 | 34 | 12 |
| 9 | 1:511 | 19.53 | 8.6 | 18.8 | 3 |
| 10 | 1:1023 | 9.77 | 9.4 | 22.2 | 2.8 |
| 11 | 1:2047 | 4.88 | 2.6 | 12.4 | 0.2 |
| -ve con | N/A | 0 | 0 | 0 | 0 |
| NTC | N/A | 0 | 0 | 0 | 0 |

C)

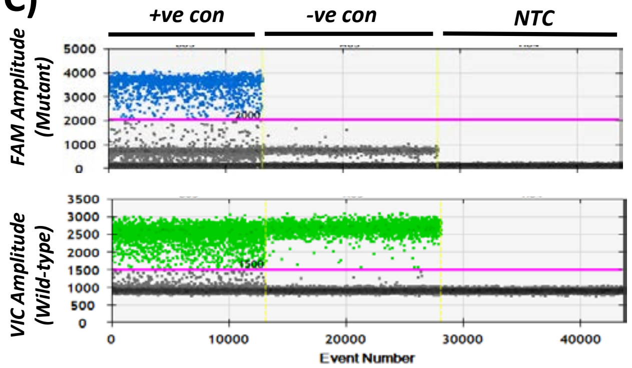


D)

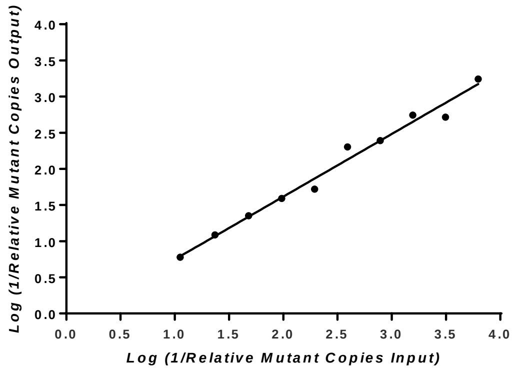


*Moe Myint et al_Supplemental Figure 7*

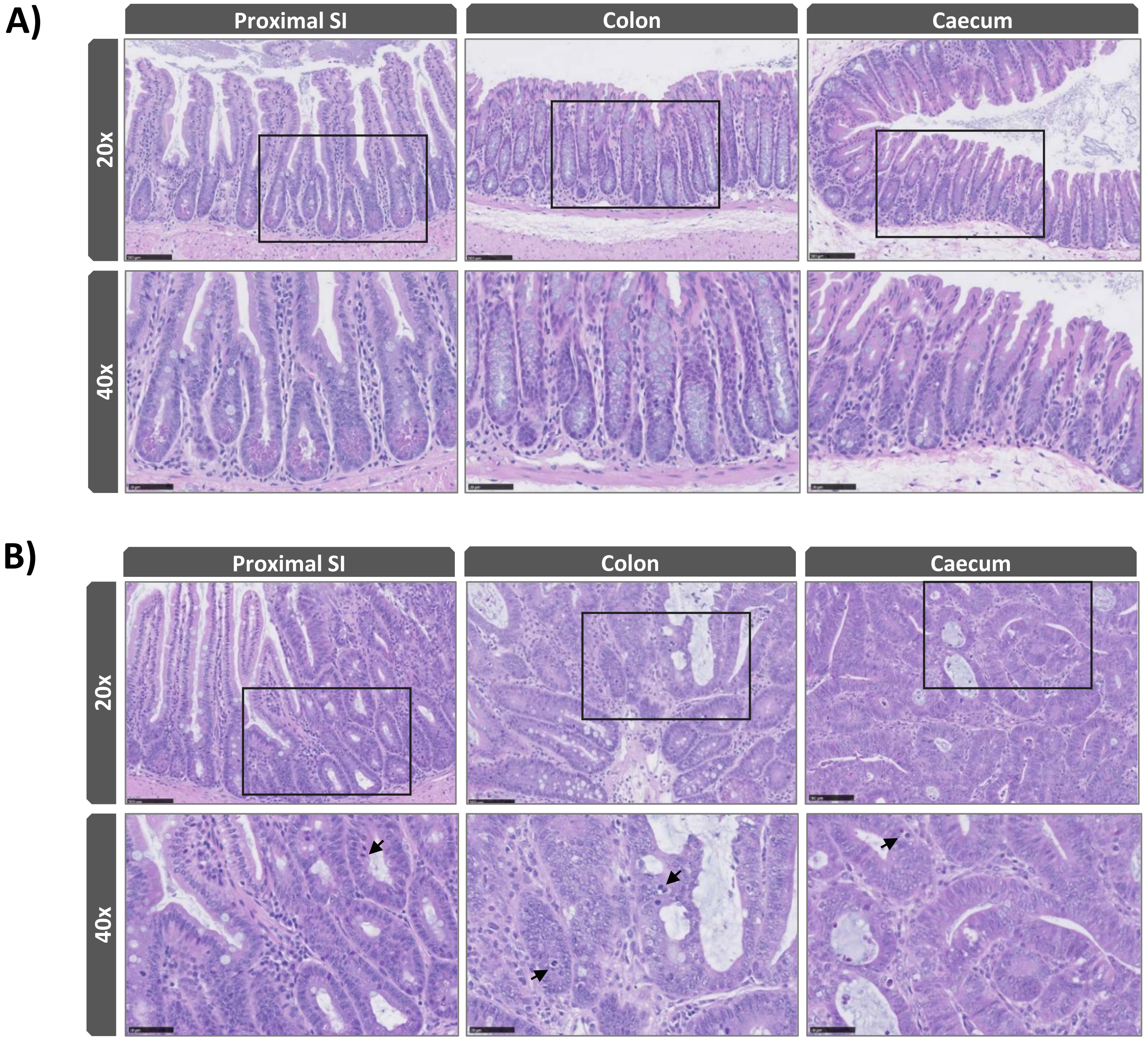


*Moe Myint et al_Supplemental Figure 8*

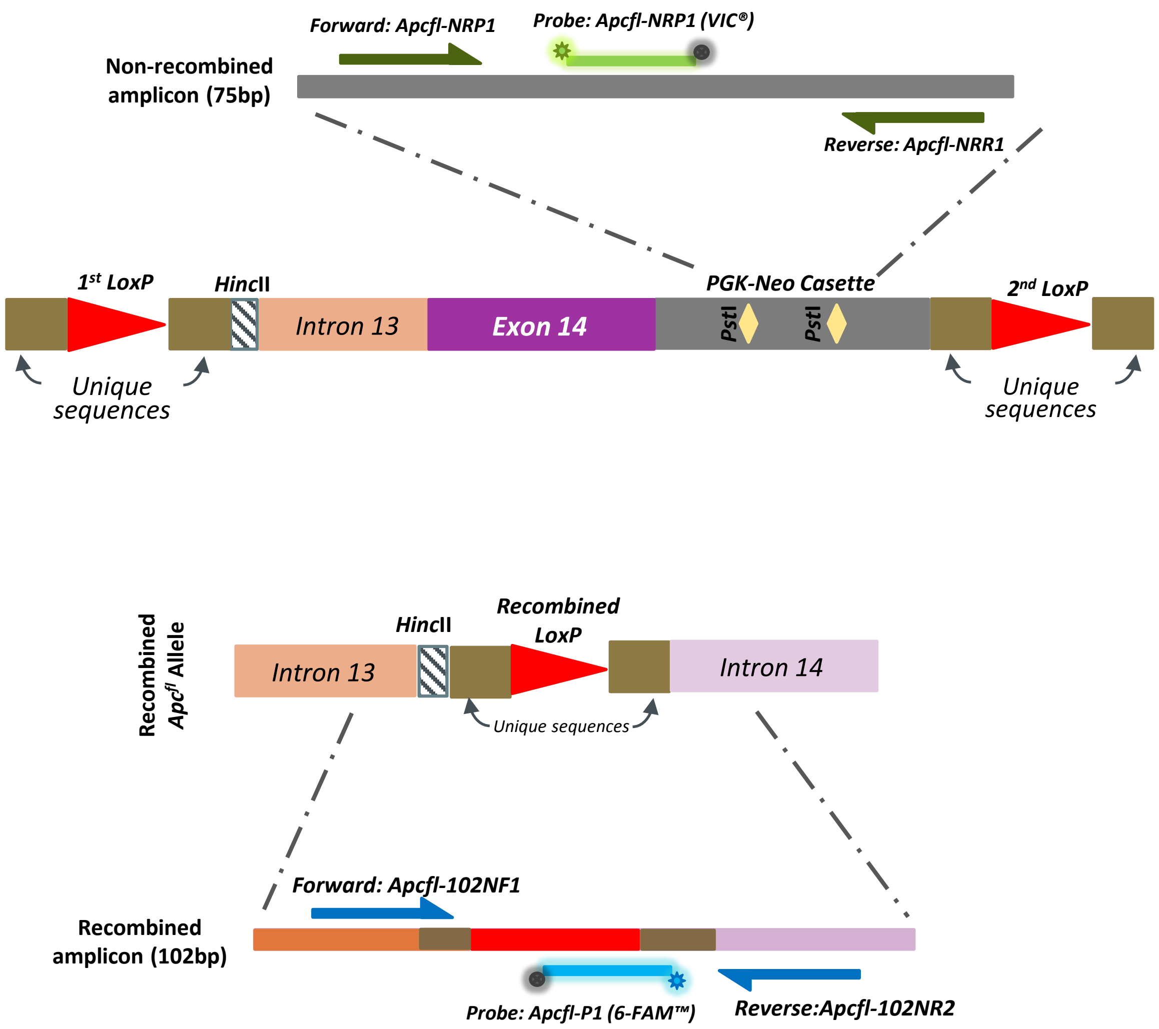


*Moe Myint et al_Supplemental Figure 9*

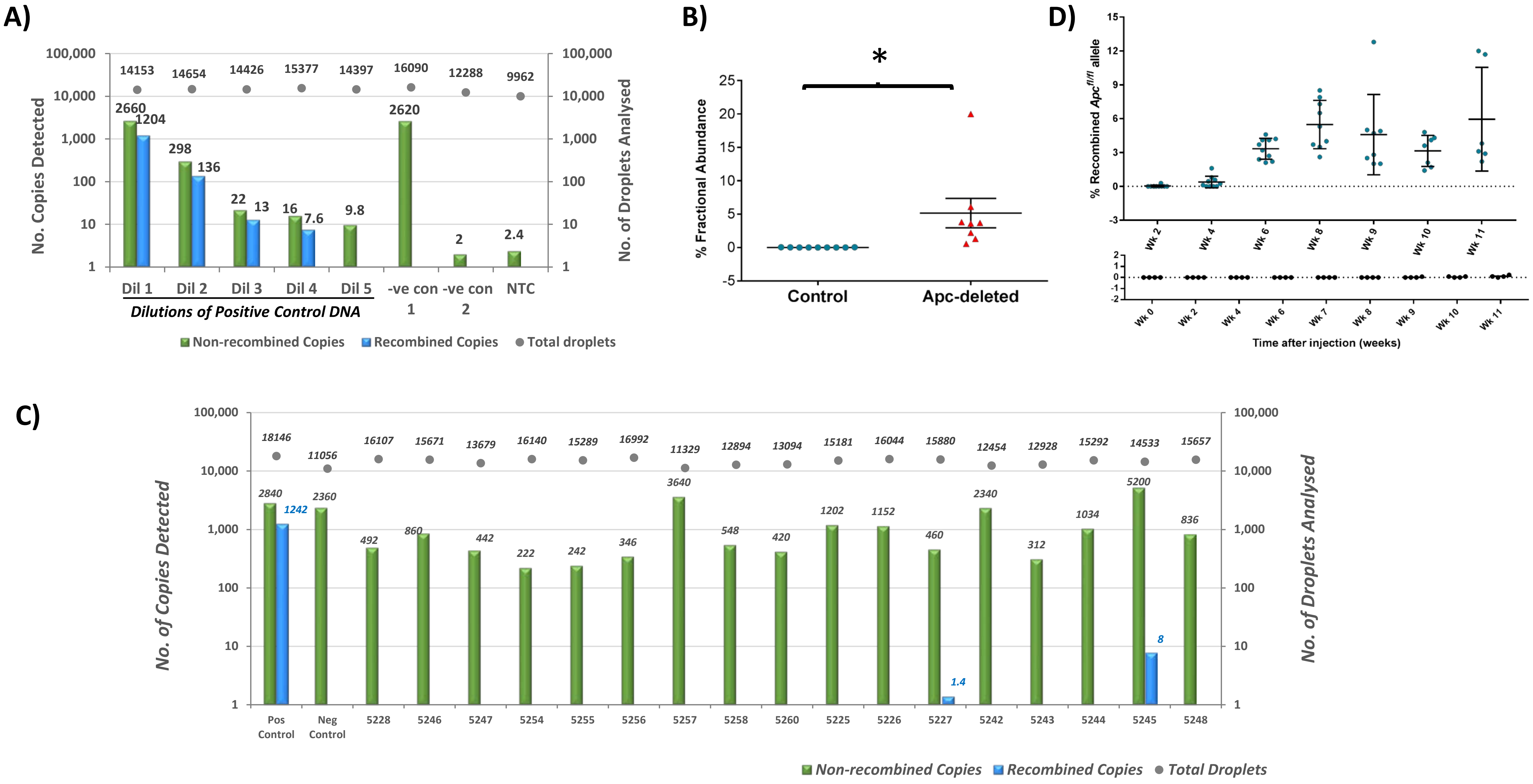


*Moe Myint et al_Supplemental Figure 10*
