# Supplementary material for: Circulating tumor DNA in patients with colorectal adenomas: assessment of detectability and genetic heterogeneity
Source: Cell Death Dis. 2018 Aug 30;9(9):894. doi: 10.1038/s41419-018-0934-x (PMC6117318; doi:10.1038/s41419-018-0934-x)
Supplement: Supplementary file 2 — Supplementary Table 1 [file 41419_2018_934_MOESM2_ESM.pptx]

## Slide 1
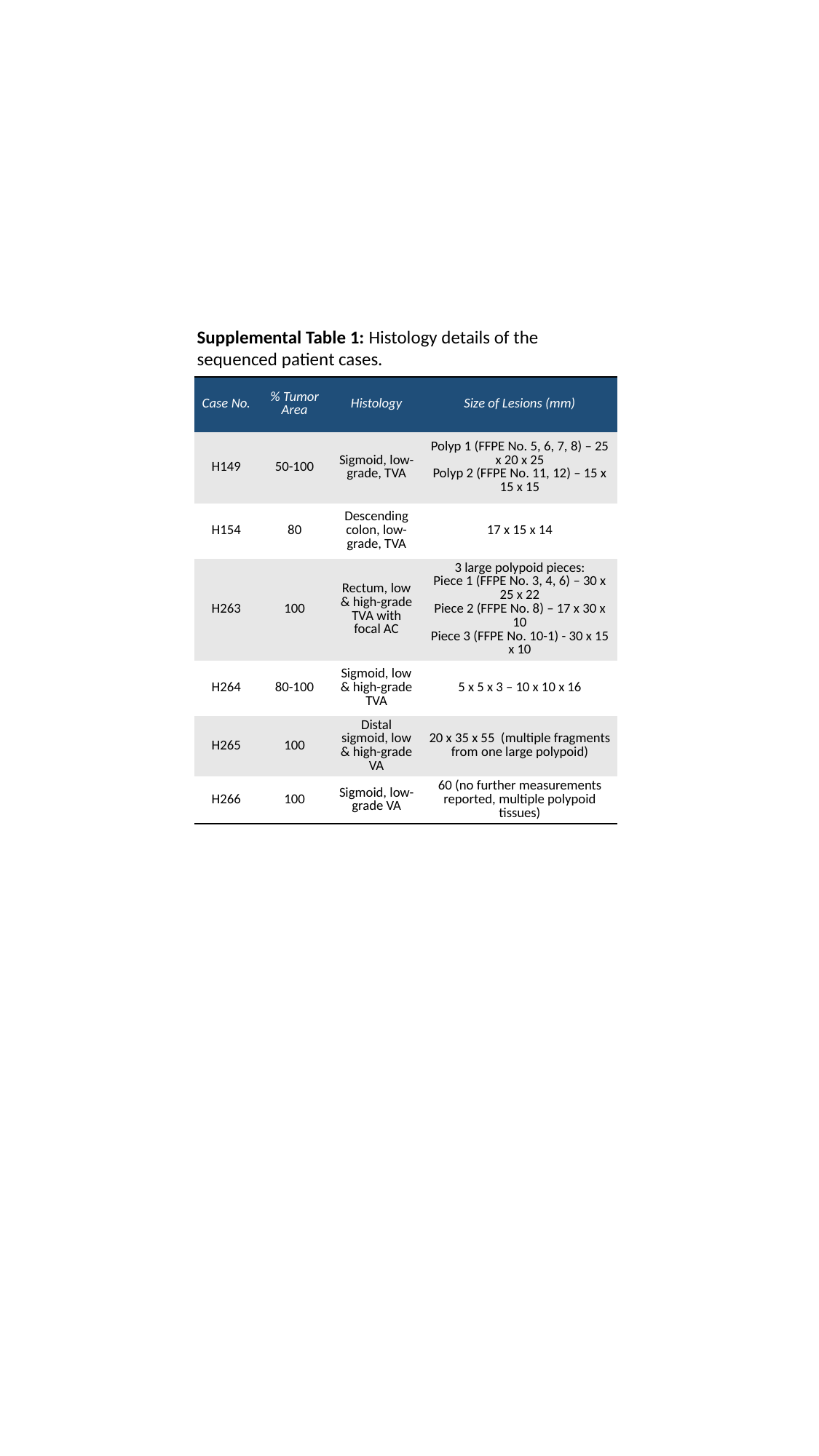

Supplemental Table 1: Histology details of the sequenced patient cases.
| Case No. | % Tumor Area | Histology | Size of Lesions (mm) |
| --- | --- | --- | --- |
| H149 | 50-100 | Sigmoid, low-grade, TVA | Polyp 1 (FFPE No. 5, 6, 7, 8) – 25 x 20 x 25 Polyp 2 (FFPE No. 11, 12) – 15 x 15 x 15 |
| H154 | 80 | Descending colon, low-grade, TVA | 17 x 15 x 14 |
| H263 | 100 | Rectum, low & high-grade TVA with focal AC | 3 large polypoid pieces: Piece 1 (FFPE No. 3, 4, 6) – 30 x 25 x 22 Piece 2 (FFPE No. 8) – 17 x 30 x 10 Piece 3 (FFPE No. 10-1) - 30 x 15 x 10 |
| H264 | 80-100 | Sigmoid, low & high-grade TVA | 5 x 5 x 3 – 10 x 10 x 16 |
| H265 | 100 | Distal sigmoid, low & high-grade VA | 20 x 35 x 55 (multiple fragments from one large polypoid) |
| H266 | 100 | Sigmoid, low-grade VA | 60 (no further measurements reported, multiple polypoid tissues) |
