# Supplementary material for: Circulating tumor DNA in patients with colorectal adenomas: assessment of detectability and genetic heterogeneity
Source: Cell Death Dis. 2018 Aug 30;9(9):894. doi: 10.1038/s41419-018-0934-x (PMC6117318; doi:10.1038/s41419-018-0934-x)
Supplement: Supplementary file 3 — Supplementary Table 2 [file 41419_2018_934_MOESM3_ESM.pptx]

## Slide 1
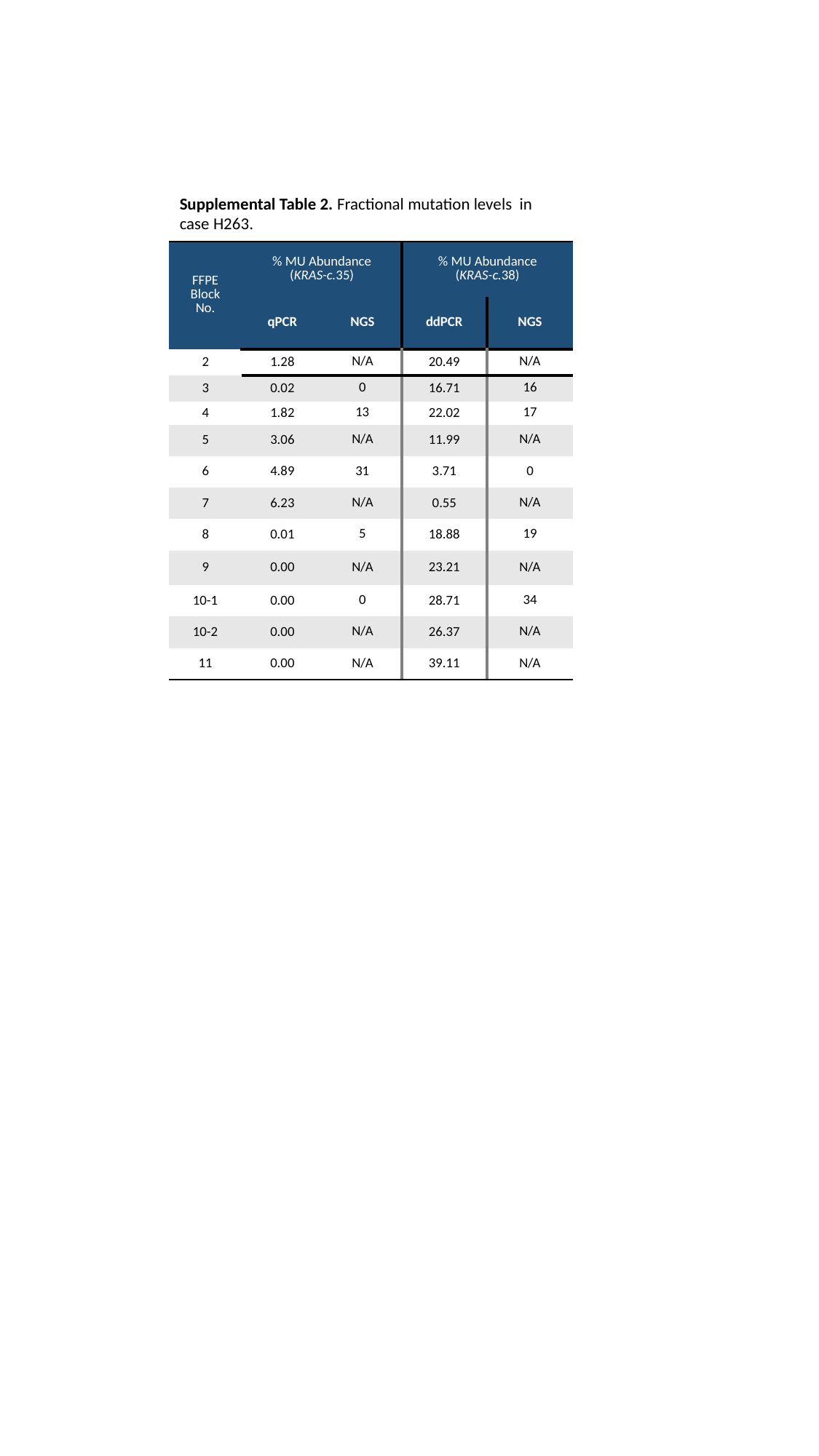

Supplemental Table 2. Fractional mutation levels in case H263.
| FFPE Block No. | % MU Abundance (KRAS-c.35) | | % MU Abundance (KRAS-c.38) | |
| --- | --- | --- | --- | --- |
| | qPCR | NGS | ddPCR | NGS |
| 2 | 1.28 | N/A | 20.49 | N/A |
| 3 | 0.02 | 0 | 16.71 | 16 |
| 4 | 1.82 | 13 | 22.02 | 17 |
| 5 | 3.06 | N/A | 11.99 | N/A |
| 6 | 4.89 | 31 | 3.71 | 0 |
| 7 | 6.23 | N/A | 0.55 | N/A |
| 8 | 0.01 | 5 | 18.88 | 19 |
| 9 | 0.00 | N/A | 23.21 | N/A |
| 10-1 | 0.00 | 0 | 28.71 | 34 |
| 10-2 | 0.00 | N/A | 26.37 | N/A |
| 11 | 0.00 | N/A | 39.11 | N/A |
