# Supplementary material for: Circulating tumor DNA in patients with colorectal adenomas: assessment of detectability and genetic heterogeneity
Source: Cell Death Dis. 2018 Aug 30;9(9):894. doi: 10.1038/s41419-018-0934-x (PMC6117318; doi:10.1038/s41419-018-0934-x)
Supplement: Supplementary file 4 — Supplementary Table 3 [file 41419_2018_934_MOESM4_ESM.pptx]

## Slide 1
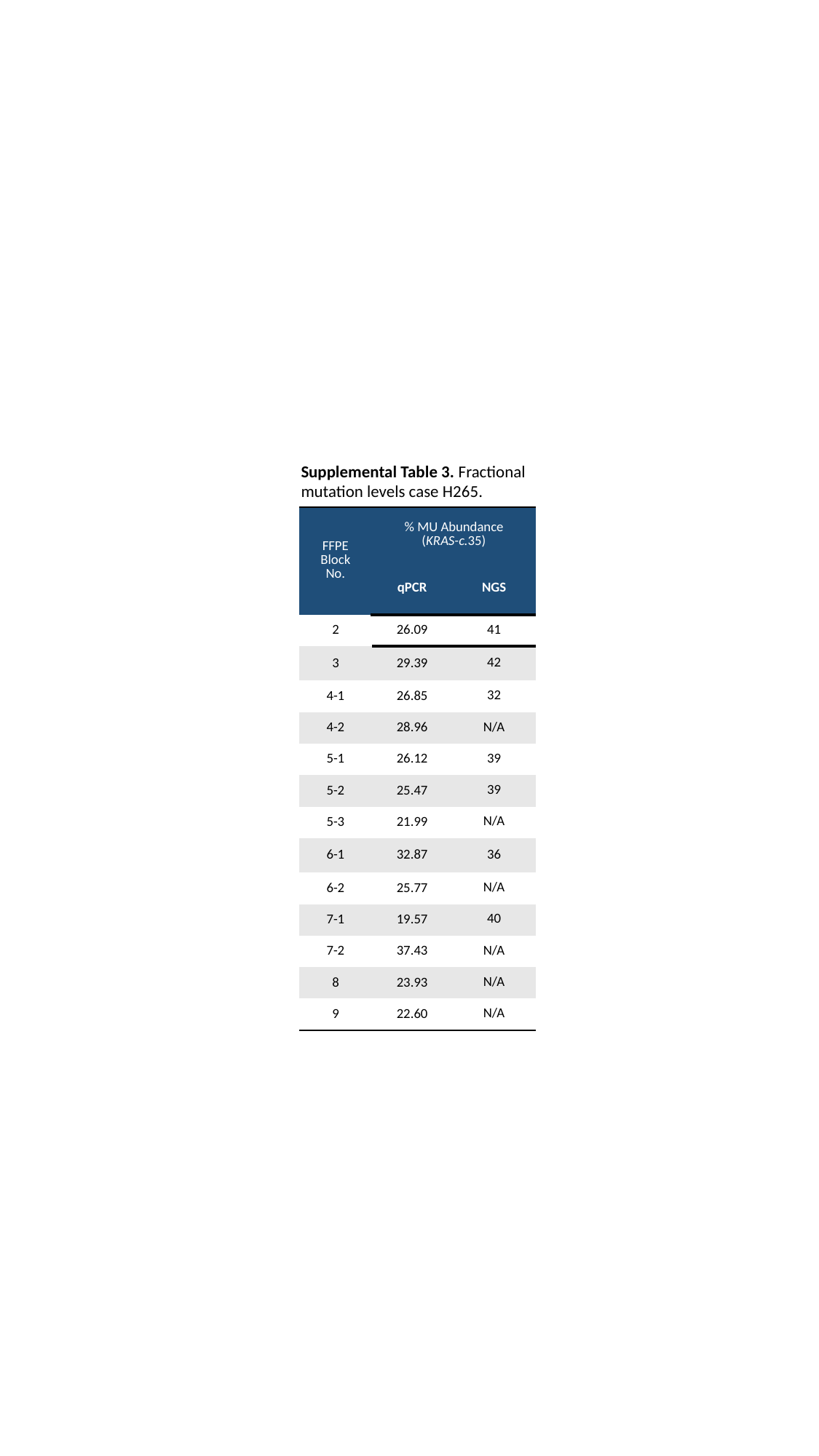

Supplemental Table 3. Fractional mutation levels case H265.
| FFPE Block No. | % MU Abundance (KRAS-c.35) | |
| --- | --- | --- |
| | qPCR | NGS |
| 2 | 26.09 | 41 |
| 3 | 29.39 | 42 |
| 4-1 | 26.85 | 32 |
| 4-2 | 28.96 | N/A |
| 5-1 | 26.12 | 39 |
| 5-2 | 25.47 | 39 |
| 5-3 | 21.99 | N/A |
| 6-1 | 32.87 | 36 |
| 6-2 | 25.77 | N/A |
| 7-1 | 19.57 | 40 |
| 7-2 | 37.43 | N/A |
| 8 | 23.93 | N/A |
| 9 | 22.60 | N/A |
