# Supplementary material for: Circulating tumor DNA in patients with colorectal adenomas: assessment of detectability and genetic heterogeneity
Source: Cell Death Dis. 2018 Aug 30;9(9):894. doi: 10.1038/s41419-018-0934-x (PMC6117318; doi:10.1038/s41419-018-0934-x)
Supplement: Supplementary file 5 — Supplementary Table 4 [file 41419_2018_934_MOESM5_ESM.pptx]

## Slide 1
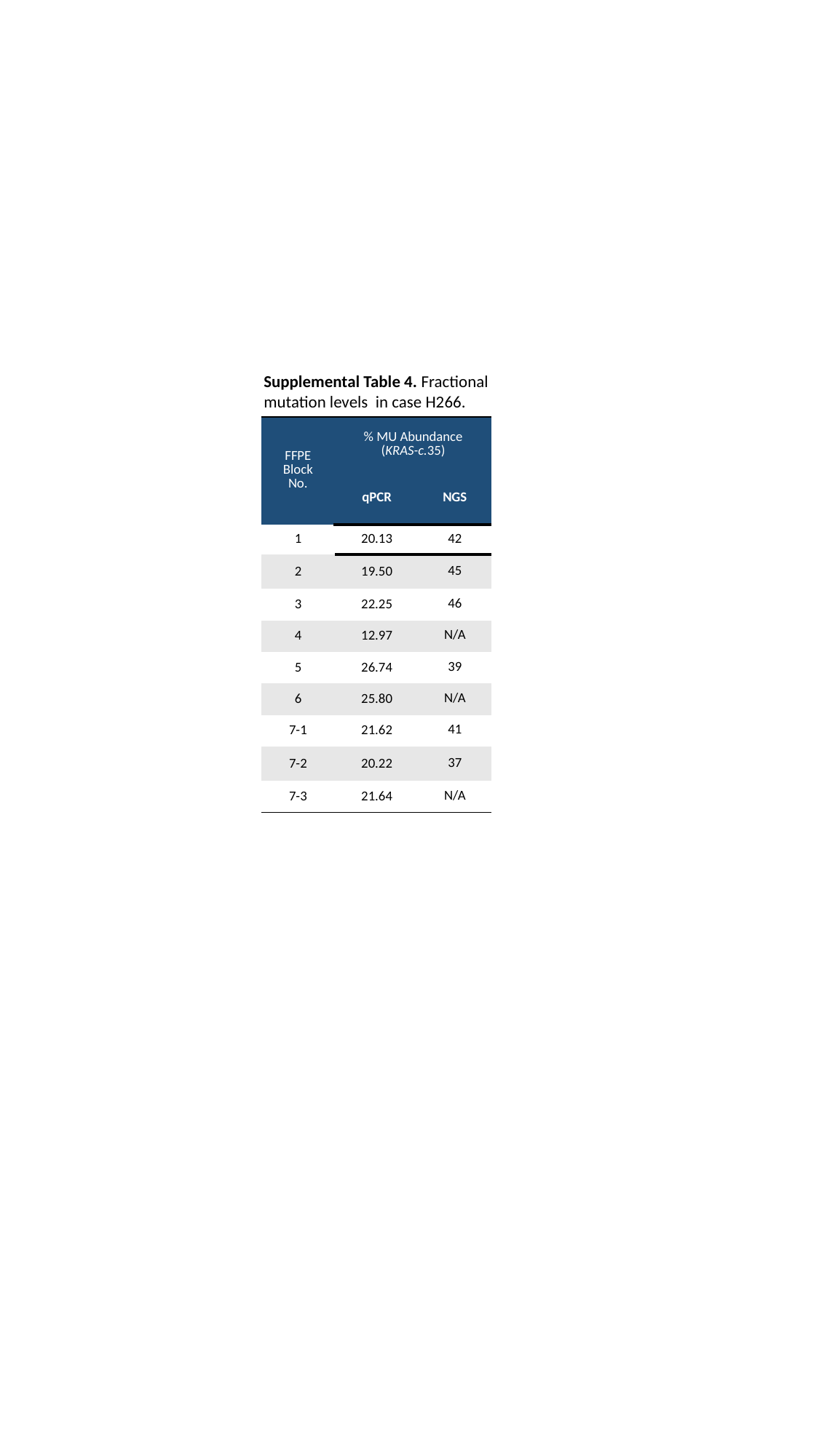

Supplemental Table 4. Fractional mutation levels in case H266.
| FFPE Block No. | % MU Abundance (KRAS-c.35) | |
| --- | --- | --- |
| | qPCR | NGS |
| 1 | 20.13 | 42 |
| 2 | 19.50 | 45 |
| 3 | 22.25 | 46 |
| 4 | 12.97 | N/A |
| 5 | 26.74 | 39 |
| 6 | 25.80 | N/A |
| 7-1 | 21.62 | 41 |
| 7-2 | 20.22 | 37 |
| 7-3 | 21.64 | N/A |
