# Supplementary material for: Circulating tumor DNA in patients with colorectal adenomas: assessment of detectability and genetic heterogeneity
Source: Cell Death Dis. 2018 Aug 30;9(9):894. doi: 10.1038/s41419-018-0934-x (PMC6117318; doi:10.1038/s41419-018-0934-x)
Supplement: Supplementary file 6 — Supplementary Table 5 [file 41419_2018_934_MOESM6_ESM.pptx]

## Slide 1
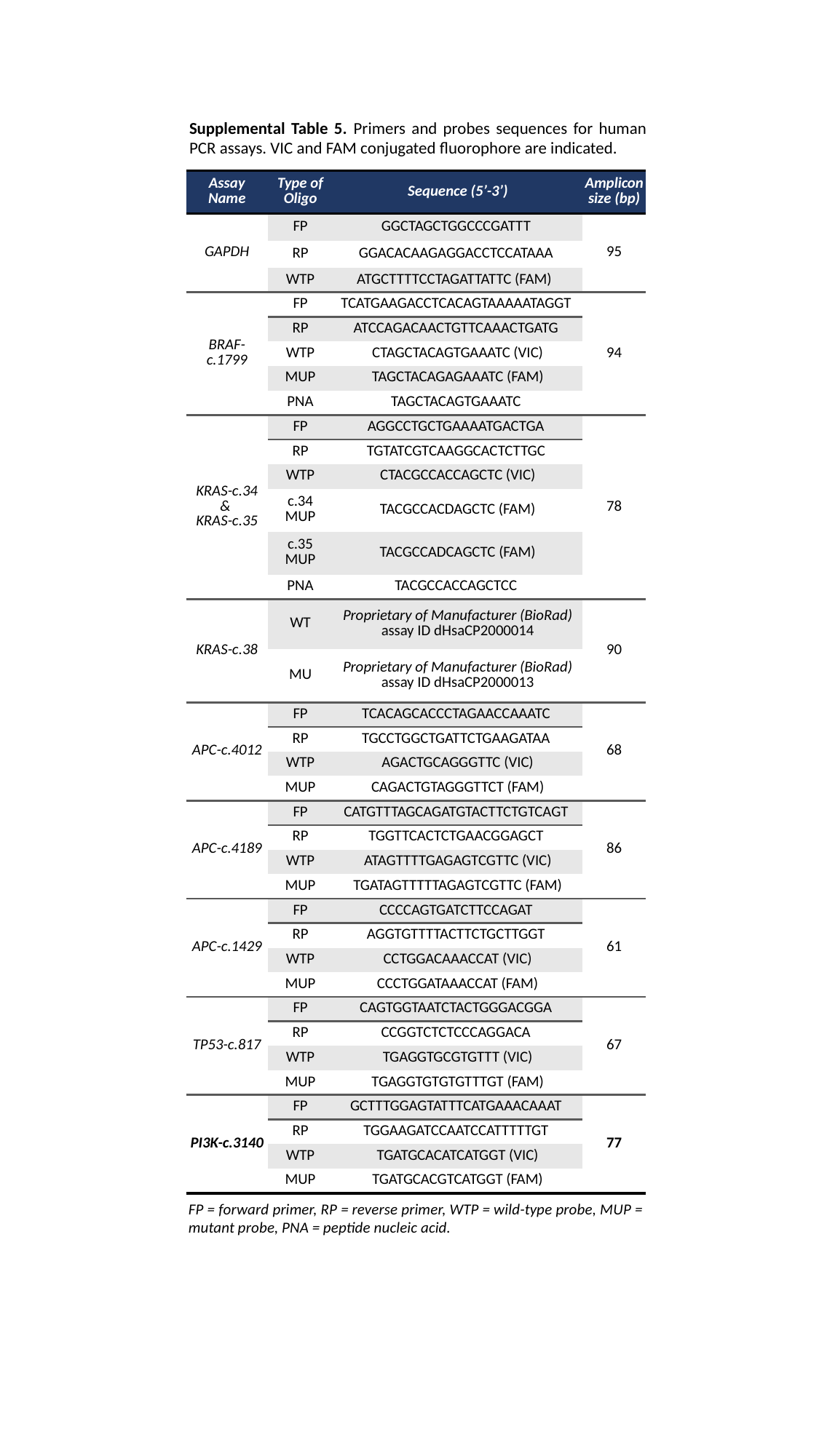

Supplemental Table 5. Primers and probes sequences for human PCR assays. VIC and FAM conjugated fluorophore are indicated.
| Assay Name | Type of Oligo | Sequence (5’-3’) | Amplicon size (bp) |
| --- | --- | --- | --- |
| GAPDH | FP | GGCTAGCTGGCCCGATTT | 95 |
| | RP | GGACACAAGAGGACCTCCATAAA | |
| | WTP | ATGCTTTTCCTAGATTATTC (FAM) | |
| BRAF-c.1799 | FP | TCATGAAGACCTCACAGTAAAAATAGGT | 94 |
| | RP | ATCCAGACAACTGTTCAAACTGATG | |
| | WTP | CTAGCTACAGTGAAATC (VIC) | |
| | MUP | TAGCTACAGAGAAATC (FAM) | |
| | PNA | TAGCTACAGTGAAATC | |
| KRAS-c.34 & KRAS-c.35 | FP | AGGCCTGCTGAAAATGACTGA | 78 |
| | RP | TGTATCGTCAAGGCACTCTTGC | |
| | WTP | CTACGCCACCAGCTC (VIC) | |
| | c.34 MUP | TACGCCACDAGCTC (FAM) | |
| | c.35 MUP | TACGCCADCAGCTC (FAM) | |
| | PNA | TACGCCACCAGCTCC | |
| KRAS-c.38 | WT | Proprietary of Manufacturer (BioRad) assay ID dHsaCP2000014 | 90 |
| | MU | Proprietary of Manufacturer (BioRad) assay ID dHsaCP2000013 | |
| APC-c.4012 | FP | TCACAGCACCCTAGAACCAAATC | 68 |
| | RP | TGCCTGGCTGATTCTGAAGATAA | |
| | WTP | AGACTGCAGGGTTC (VIC) | |
| | MUP | CAGACTGTAGGGTTCT (FAM) | |
| APC-c.4189 | FP | CATGTTTAGCAGATGTACTTCTGTCAGT | 86 |
| | RP | TGGTTCACTCTGAACGGAGCT | |
| | WTP | ATAGTTTTGAGAGTCGTTC (VIC) | |
| | MUP | TGATAGTTTTTAGAGTCGTTC (FAM) | |
| APC-c.1429 | FP | CCCCAGTGATCTTCCAGAT | 61 |
| | RP | AGGTGTTTTACTTCTGCTTGGT | |
| | WTP | CCTGGACAAACCAT (VIC) | |
| | MUP | CCCTGGATAAACCAT (FAM) | |
| TP53-c.817 | FP | CAGTGGTAATCTACTGGGACGGA | 67 |
| | RP | CCGGTCTCTCCCAGGACA | |
| | WTP | TGAGGTGCGTGTTT (VIC) | |
| | MUP | TGAGGTGTGTGTTTGT (FAM) | |
| PI3K-c.3140 | FP | GCTTTGGAGTATTTCATGAAACAAAT | 77 |
| | RP | TGGAAGATCCAATCCATTTTTGT | |
| | WTP | TGATGCACATCATGGT (VIC) | |
| | MUP | TGATGCACGTCATGGT (FAM) | |
FP = forward primer, RP = reverse primer, WTP = wild-type probe, MUP = mutant probe, PNA = peptide nucleic acid.
