# Supplementary material for: Circulating tumor DNA in patients with colorectal adenomas: assessment of detectability and genetic heterogeneity
Source: Cell Death Dis. 2018 Aug 30;9(9):894. doi: 10.1038/s41419-018-0934-x (PMC6117318; doi:10.1038/s41419-018-0934-x)
Supplement: Supplementary file 7 — Supplementary Table 6 [file 41419_2018_934_MOESM7_ESM.pptx]

## Slide 1
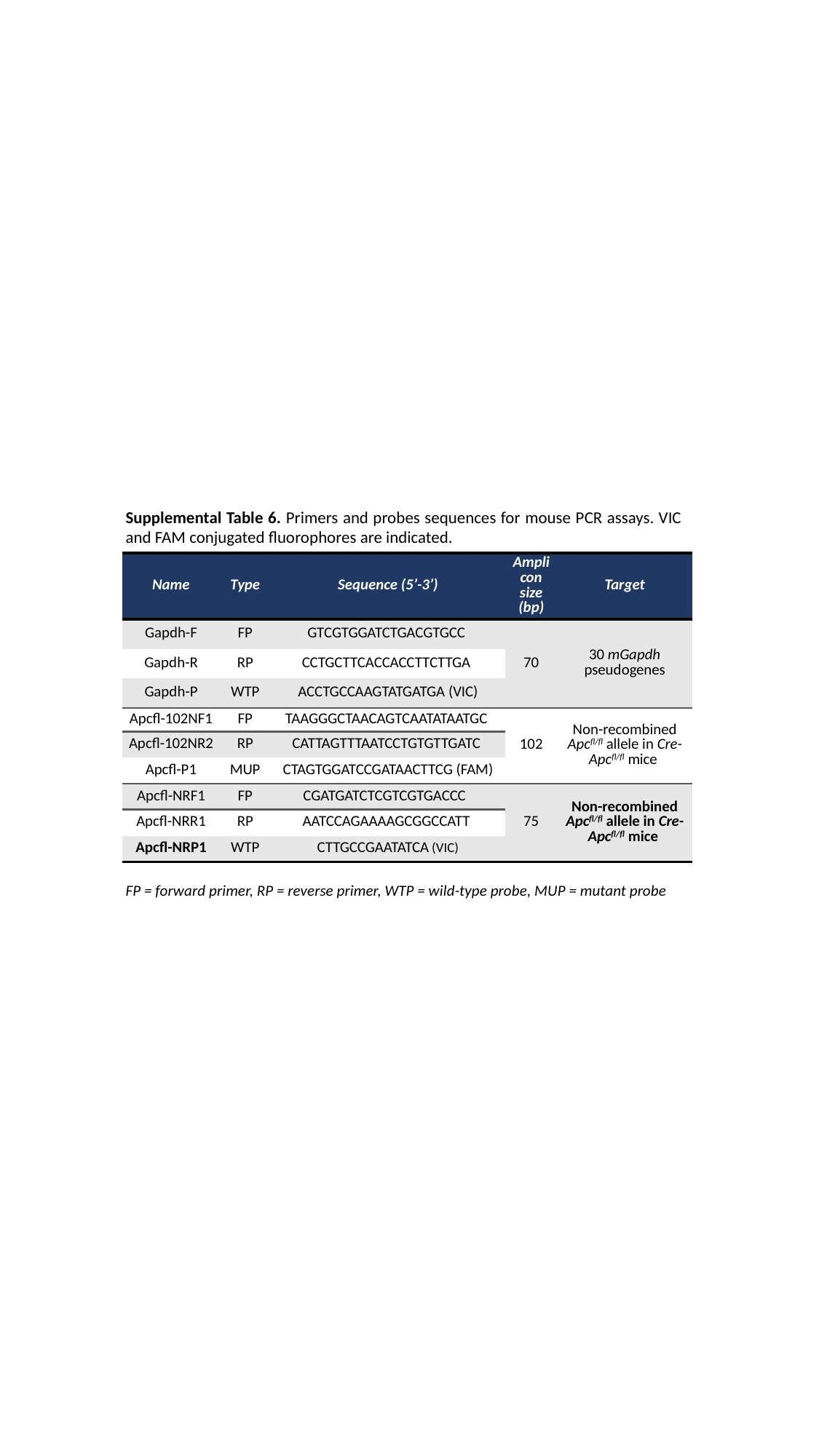

Supplemental Table 6. Primers and probes sequences for mouse PCR assays. VIC and FAM conjugated fluorophores are indicated.
| Name | Type | Sequence (5’-3’) | Amplicon size (bp) | Target |
| --- | --- | --- | --- | --- |
| Gapdh-F | FP | GTCGTGGATCTGACGTGCC | 70 | 30 mGapdh pseudogenes |
| Gapdh-R | RP | CCTGCTTCACCACCTTCTTGA | | |
| Gapdh-P | WTP | acctgccaagtatgatga (VIC) | | |
| Apcfl-102NF1 | FP | TAAGGGCTAACAGTCAATATAATGC | 102 | Non-recombined Apcfl/fl allele in Cre-Apcfl/fl mice |
| Apcfl-102NR2 | RP | CATTAGTTTAATCCTGTGTTGATC | | |
| Apcfl-P1 | MUP | CTAGTGGATCCGATAACTTCG (FAM) | | |
| Apcfl-NRF1 | FP | CGATGATCTCGTCGTGACCC | 75 | Non-recombined Apcfl/fl allele in Cre-Apcfl/fl mice |
| Apcfl-NRR1 | RP | AATCCAGAAAAGCGGCCATT | | |
| Apcfl-NRP1 | WTP | CTTGCCGAATATCA (VIC) | | |
FP = forward primer, RP = reverse primer, WTP = wild-type probe, MUP = mutant probe
