# Supplementary material for: Circulating tumor DNA in patients with colorectal adenomas: assessment of detectability and genetic heterogeneity
Source: Cell Death Dis. 2018 Aug 30;9(9):894. doi: 10.1038/s41419-018-0934-x (PMC6117318; doi:10.1038/s41419-018-0934-x)
Supplement: Supplementary file 8 — Supplementary figure legends [file 41419_2018_934_MOESM8_ESM.docx]

**Supplemental figure legends**

**Supplemental Figure 1. Plasma cfDNA Analysis Using the *BRAF-c.1799* Assay. A)** Merged data from two runs of the *BRAF-c.1799* assay. The number of WT (green bars) and mutant (blue bars) copies detected are shown on left y-axis. The total number of droplets analyzed (grey dots) are indicated on right y-axis. “+ve con” indicates positive control from 10ng of genomic DNA isolated from HT-29 CRC cell line. “–ve con” indicates negative control from 10ng of hgDNA. NTC = no template control. **B)** Tabulated data of patient samples tested. Histology data and polyp dimensions were obtained from histology reports. HP = hyperplastic polyp, LG = low-grade, VA = villous adenoma.

**Supplemental Figure 2. Validation of *BRAF* and *KRAS* Assays on ddPCR.** Bar Graphs for **A)** *BRAF-c.1799*, **B)** *KRAS-c.34*, **C)** *KRAS-c35*, **D)** *KRAS-c.38* mutation assays by ddPCR. Dilutions (Dil) 1 to 6 represent reactions with decreasing mutant DNA quantity with increasing WT background in 10ng of total DNA. WT copies (green bars) and mutant copies (blue bars) detected were plotted on the left y-axis. Right y-axis showed the number of droplets analyzed (grey dots). Negative control (-ve con) was hgDNA. NTC = no template control. Positive mutant DNA was isolated from colorectal cancer cell lines HT-29 (*BRAF c.1799T>A*), SW480 (*KRAS* c.35G>T) and HCT116 (*KRAS* c.38G>A), and lung cancer cell line A549 (*KRAS c.34G>A*). **E)** 1D ddPCR plot showing the indicated ddPCR assays tested on the following samples: plasma, 2.5ng ctDNA isolated from a patient with a stage 4 *BRAF-c.1799*-mutant CRC; +ve-1, 10ng DNA isolated from RKO cell lines (*BRAF c.1799T>A);* +ve-2, 10ng ctDNA-like SeraCare positive control (contains *BRAF-c.1799 and KRAS-c.35* at 1% fractional abundance); +ve-3, 10ng DNA isolated from *KRAS-c.35*-mutant tumor tissue, -ve, NTC, no template control.

**Supplemental Figure 3. Validation of *APC-c.4012* Assay on ddPCR. A)** DNA extracted from the sequenced FFPE sample (H266) was used as positive control (average mutant allele frequency: 45.5%). Positive control was diluted in 1:2 ratio serially in excess of hgDNA as background. Dilutions (Dil) 1 to 11 represent reactions with decreasing mutant DNA quantity. WT (green bars) and mutant copies (blue bars) detected were plotted on the left y-axis. Right y-axis showed the number of droplets analyzed (grey dots). Negative control (-ve con) was hgDNA. NTC = no template control. **B)** Poisson distribution analysis for the mutant target detection. **C)** 1D ddPCR plots showing manual threshold gating (pink lines) for the mutant (FAM) and WT (VIC) targets. The positive control (+ve con) was Dil 1. **D)** Linear regression analysis of mutant copies input (x-axis) vs. mutant copies detected (y-axis).The line has the equation Y=0.9796X-0.4992 with the R^2^ value of 0.9864.

**Supplemental Figure 4. Validation of *APC-c.4285* Assay on ddPCR. A)** DNA extracted from the sequenced FFPE sample (H263) was used as a positive control (average allele frequency: 44.4%). Positive control was diluted in 1:2 ratio serially in excess of hgDNA. Dilutions (Dil) 1 to 11 represent reactions with decreasing mutant DNA quantity. WT (green bars) and mutant copies (blue bars) detected were plotted on the left y-axis. Right y-axis showed the number of droplets analyzed (grey dots) for each reaction. Negative control (-ve con) was hgDNA. NTC = no template control. **B)** Poisson distribution analysis for the mutant target detection. **C)** 1D ddPCR plots showing manual threshold gating (pink lines) for the mutant (FAM) and WT (VIC) targets. The positive control (+ve con) was Dil 1. **D)** Linear regression analysis of mutant copies input (x-axis) vs. mutant copies detected (y-axis). The line has the equation Y=0.9548X-0.3964 with the R^2^ value of 0.9706.

**Supplemental Figure 5. Validation of *APC-c.4189* Assay on ddPCR. A)** DNA extracted from sequenced FFPE sample (H266) was used as a positive control (average mutant allele frequency: 52.5%). Positive control was diluted in 1:2 ratio serially in excess of hgDNA as background. Dilutions (Dil) 1 to 11 represent reactions with decreasing mutant DNA quantity. WT (green bars) and mutant copies (blue bars) detected were plotted on the left y-axis. Right y-axis showed the number of droplets analyzed (grey dots) for each reaction. Negative control (-ve con) was hgDNA. NTC = no template control. Both the negative control and NTC showed contamination. **B)** Poisson distribution analysis for the mutant target detection. **C)** 1D ddPCR plots showing manual threshold gating (pink lines) for the mutant (FAM) and WT (VIC) targets. The positive control (+ve con) was Dil 1. **D)** Linear regression analysis of mutant copies input (x-axis) vs. mutant copies detected (y-axis). The line has the equation Y=0.8704X-0.3201 with the R^2^ value of 0.9672.

**Supplemental Figure 6. Validation of *TP53-c.817* Assay on ddPCR. A)** DNA extracted from the sequenced FFPE sample (H265) was used as a positive control (average mutant allele frequency: 43.0%). Positive control was diluted in 1:2 ratio serially in excess of hgDNA. Dilutions (Dil) 1 to 11 represent reactions with decreasing mutant DNA quantity. WT (green bars) and mutant copies (blue bars) detected were plotted on the left y-axis. Right y-axis showed the number of droplets analyzed (grey dots). Negative control (-ve con) was hgDNA. NTC = no template control. **B)** Poisson distribution analysis for mutant target detection. **C)** 1D ddPCR plots showing manual threshold gating (pink lines) for the mutant (FAM) and WT (VIC) targets. The positive control (+ve con) was Dil 1. **D)** Linear regression analysis of mutant copies input (x-axis) vs. mutant copies detected (y-axis).The line has the equation Y=0.9035X-0.4723 with the R^2^ value of 0.9965.

**Supplemental Figure 7. Validation of *PI3K-c.3140* Assay on ddPCR. A)** DNA extracted from sequenced FFPE sample (H263) was used as a positive control (average mutant allele frequency: 9.6%). Positive control was diluted in 1:2 ratio serially in hgDNA as background. Dilutions (Dil) 1 to 11 represent reactions with decreasing mutant DNA quantity. WT (green bars) and mutant copies (blue bars) detected were plotted on the left y-axis. Right y-axis showed number of droplets analyzed (grey dots) for each reaction. Negative control (-ve con) was hgDNA. NTC = no template control. **B)** Poisson distribution analysis for the mutant target detection. **C)** 1D ddPCR plots showing manual threshold gating (pink lines) for the mutant (FAM) and WT (VIC) targets. The positive control (+ve con) was Dil 1. **D)** Linear regression analysis of mutant copies input (x-axis) vs. mutant copies detected (y-axis). The line has the equation Y=0.8671X-0.1187 with the R^2^ value of 0.982.

**Supplemental Figure 8. Histology of Control and *Apc*-deleted Mice.** Hematoxylin and eosin stained images of proximal small intestine, colon and caecum. Insets in the upper panels (20x, scale bar= 100μm) marked the regions magnified in the lower panels (40x, scale bar= 50μm). **A)** A representative control mouse shows a normal epithelium with organized crypts and villi. **B)** A representative example of *Apc*-deleted mouse (ID5243) with several adenomas along the gastrointestinal tract. Mitotic cells are indicated by black arrows (40x). Size bars = 100μm in 20x and 50μm in 40x images.

**Supplemental Figure 9. Assay Design for Detection of the *Apc^fl/fl^* Alleles** Schematic representation of the primers and probes locations for the PCR-enabled detection of the non-recombined (above) and recombined (below) *Apc^fl/fl^* allele. See additional methods for details.

**Supplemental Figure 10. Analysis of *Apc^fl/fl^* Allele.** In house developed assays for the detection of recombined and non-recombined mouse *Apc^fl/fl^* alleles were tested on ddPCR platform. **A)** Assessment of the ddPCR assay efficiency for the *Apc* allele assays. Graph shows the numbers of recombined (blue) or non-recombined (green) copies detected on the left y-axis, and the number of droplets analyzed (grey dots) on the right y-axis. Positive control was intestinal tissue DNA from an *Apc*-deleted mouse. The total amount of DNA was kept constant at 10ng for all five dilutions (Dil 1-5) tested. Negative control 1 (-ve con 1, i.e. non-recombined allele) was 10ng of DNA isolated from an ear biopsy from the same mouse. Negative control 2 (-ve con 2, i.e. without floxed *Apc* alleles) was 10ng commercially available mouse genomic DNA. NTC = no template control. **B)** Percentage fractional abundances of recombined alleles from small intestinal tissue from the control (blue) and *Apc*-deleted (red) mice. Each dot represents one mouse; error bars represent mean ±SEM. P<0.05, two-tailed t-test, n=9 for controls and 8 for *Apc*-deleted mice. **C)** Graph showing the numbers of non-recombined (green bars) and recombined (blue bars) copies detected in the plasma of control and *Apc*-deleted mice (left y-axis). Number of droplets analyzed (grey dots) for each samples is shown on right y-axis. n=9 for controls and 8 for *Apc*-deleted mice. **D)** Fractional abundance of the *Apc* recombined allele in a time course analysis of stool DNA collected after tamoxifen-induced *Apc* deletion. Each dot represents one mouse. Error bars represent mean ±SD. n=4 for controls and n=6-10 for *Apc*-deleted mice. All control mice (black dots) were negative for the recombined alleles.
